# Material insecurity and religiosity: A causal analysis

**DOI:** 10.1017/ehs.2023.29

**Published:** 2024-01-17

**Authors:** Benjamin Grant Purzycki, Theiss Bendixen

**Affiliations:** Aarhus University, Denmark

**Keywords:** religiosity, education, material security, causal inference

## Abstract

Some cultural evolutionary models predict that under stressful reductions of well-being, individuals will be more attracted and fastidiously adhere to traditional systems of norms that promote solidarity and cooperation. As religious systems can bolster human relationships with a variety of mechanisms, the material insecurity hypothesis of religion posits that individual religiosity will increase under conditions of material insecurity. The bulk of the literature up to this point has been correlational and cross-national. Here, across 14 field sites, we examine the causal role that educational attainment and food insecurity play in religiosity. We find that years of formal education and food insecurity do not consistently contribute to individual religiosity cross-culturally. We conclude with a discussion of some theoretical and methodological implications. As a general workflow for cross-cultural causal research in the quantitative social sciences, the present work is a modest but necessary first step in reliably estimating causation in the material insecurity hypothesis of religiosity.

## Graphical abstract

**Figure:**
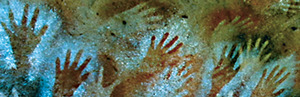

**Social media summary:** This report finds that education or food security play no cross-culturally robust causal role in individual religiosity.

## 1. Introduction

Some models of cultural evolutionary processes posit that under stressful conditions, individuals will be attracted to and punish violations of cooperative norms (Roos et al., 2015). As a cultural system that bolsters cooperation (Lang et al., 2019; Sosis & Bressler, 2003; Sosis & Ruffle, 2003; cf. Major-Smith, 2023), we should expect to see that stress and insecurity increase religiosity, defined here as the mental and behavioural commitment to traditions and beliefs associated with spiritual agents. The bulk of the literature assessing this hypothesis typically exploits nation-level data from fully market-integrated societies and has yet to embrace formal causal inference methods. This report introduces causal inference to the material insecurity hypothesis literature and does so by applying it to individual-level data collected across 14 field sites from around the world.

## 2. Theory

### 2.1. Cultural evolution of commitment

Formal evolutionary game theoretical models show that increased threats to individual well-being contribute to the evolution of cooperative norm strength and policing; sudden reductions in within-group, individual payoffs induce stronger norm adherence and favour more costly punishment of norm violators (Roos et al., 2015). Empirical evidence is consistent with these models; cross-culturally, such ‘cultural tightness’ corresponds to various threats to well-being (Gelfand et al., 2011; Jackson et al., 2020). In general, then, it follows that under threatening conditions, individuals will flock towards and adhere more strongly to cultural systems that are especially adept at alleviating such stress.

As a social system that can (a) soothe anxiety (Lang et al., 2015; Pargament et al., 1998; Sosis, 2007; Sosis & Handwerker, 2011), (b) bolster within-group cooperation and solidarity (Lang et al., 2019; Sosis & Bressler, 2003; Sosis & Ruffle, 2003), (c) flexibly attend to novel perturbations in and threats to cooperation (Purzycki et al., 2020, 2022a; cf. McNamara et al., 2016) and (d) include beliefs for the punishment of violations of moral and other social norms (Johnson, 2005, 2016; Norenzayan, 2013; Purzycki et al., 2022c; Stark, 2001), religion should be especially attractive for individuals enduring insecurity and stress. In other words, under duress, individuals are likely to intensify their commitment to the kinds of traditional norms and behaviours that have served prior generations and facilitated the kinds of social support that bolsters one's survival and reproduction, and religion has been shown to be especially supportive of these traits (Henrich et al., 2019; Inglehart, 2020; Purzycki & Sosis, 2022; Sibley & Bulbulia, 2012). As stressed by Inglehart (2020), the kinds of existential security that religion might uphold are both physical and intellectual (i.e. religious worldviews provide answers to life's bigger questions). We focus here on the former, hence, the ‘material insecurity hypothesis’.

### 2.2. Material insecurity hypothesis

While not without complications, a wide-ranging sociological literature is consistent with the material insecurity hypothesis (cf. Stark, 1999). Yet a host of socio-demographic factors associated with material insecurity also appear to increase religiosity at the individual and group levels (see Storm, 2017). For example, food insecurity, poverty and wealth inequality have been found to co-vary with religiosity (Baimel et al., 2022; Hekmatpour, 2020; Höllinger & Muckenhuber, 2019; Inglehart, 2020; Norris & Inglehart, 2011; Purzycki et al., 2018b; Ruiter & Van Tubergen, 2009; Solt et al., 2011), but as we are presently interested in assessing whether or not food security *causes* changes in religiosity, we must declare a particular causal direction. Furthermore, in addition to the predicted rise in religiosity owing to insecurity associated with age and proximity to death (Jong & Halberstadt, 2018), some cross-cultural evidence suggests that important indices of religious commitment can change across the lifespan in important ways (Bengtson et al., 2015; Purzycki & Bendixen, 2020; Shaver & Sosis, 2014).

Additionally, sex is variously associated with religiosity (Schnabel et al., 2018; Vardy et al., 2022; Walter & Davie, 1998) as are fertility and family size (Blume, 2009; Glavatskaya et al., 2018; Inglehart, 2020; Shaver et al., 2020). On evolutionary theoretical grounds, family size in particular has a complicated relationship with material insecurity (Lawson et al., 2012; Purzycki et al., 2018b; Strassmann & Gillespie, 2002), but evidence points to a relationship nonetheless. Similarly, education level is directly implicated in the number of children one has (Becker et al., 2010; Goodman et al., 2012) and shows a widely touted – but no less complicated – relationship with security and well-being (see Boarini & Strauss, 2010; Desjardins, 2008; Giambona et al., 2022).

In addition to its role in material security, educational attainment is also of particular interest in the study of religion. Exposure to formal, secular education has long been asserted to weaken religious faith owing to the cultivation of critical thinking skills (Dawkins, 2016; Weber, 1993 [1920]). In other words, educational attainment might both directly and indirectly decrease religiosity. However, while some cross-national assessments do indeed show negative associations between years of formal education and religiosity (e.g. Inglehart, 2020; Norris & Inglehart, 2011, pp. 271–274), others reveal that both the magnitude and even the direction of the estimated association exhibits substantial cross-cultural variability (e.g. Albrecht & Heaton, 1984; Pew Research Center, 2016; Ruiter & Van Tubergen, 2009; Schwadel, 2011, 2015).

As insightful as this literature is, it has some important limitations worth addressing. Theoretically, despite the explicit causal language across the theories that motivate this work, there is a distinct lack of causal modelling in the literature. This makes it difficult to synthesise various works into a single causal framework. Indeed, the empirical efforts are primarily correlational and do not formally model the manifold causal pathways by which education and food security could affect individual religious commitments. So, while theory is causal, the models are often informal and analyses typically focus on significant correlations rather than counterfactual states.

In terms of data, the bulk of this work often focuses on group-level relationships. Yet, group-level correlations can mask or muddle individual-level effects (Robinson, 2009; Simpson, 1951; Storm, 2017; see Supporting Information Section 2 of the present report for further discussion and demonstration of this point). As such, individual-level, high-resolution data are important for adequately assessing individual-level hypotheses. In terms of sampling, most of the data that populates these studies are collected among individuals in fully market-integrated industrialised societies. Given the variation in food security and access to education the world exhibits, we should be able to see the anticipated effects of these indices of material security on religiosity across the range of communities. Here, we offer a modest step towards rectifying these issues by (a) synthesising a host of hypotheses into a single causal model, (b) being explicit about and simulating the assumptions of our model, (c) applying this model to data sampled from a diverse range of societies and (d) attending to individual-level effects using a general and principled causal inference method.

## 3. Assessing causation

### 3.1. Causal inference

Across academic fields, there is increasing recognition that (a) causal explanatory accounts of natural phenomena require explicitly derived models and (b) estimating causation is best approached from an interventionist perspective (variously known as the *manipulationist*, *counterfactual* or *potential outcomes* frameworks; Hernan & Robins, 2020; Morgan & Winship, 2015; Pearl et al., 2016; Woodward, 2005). In this view, establishing causation requires that we not only examine what our data tell us about worlds that exist, but also form deliberately-calculated inferences of causal relationships derived from worlds that *would* exist, given particular conditions.

Quite often, the standard procedure of including a statistical model with all variables under consideration simply assumes that all variables are predictive of the dependent variable and ignores any internal causal structure. Yet, ‘controlling’ for everything can actually create more problems than it solves because otherwise ignored causal relationships that are internal to those predictors can introduce further confounding effects (e.g. conditioning on a common descendant of the exposure and outcome – *collider bias*) or suppress actual causal effects (e.g. conditioning on a mediator or a post-treatment variable). Put simply, under some conditions, holding some variables constant (i.e. controlling) can be inconsequential, while under others it can be disastrous to inference (Achen, 2005; Cinelli et al., 2020; Westreich & Greenland, 2013; Wysocki et al., 2022). The conditions that matter here are the causal structure of variables germane to testing hypotheses. Making these conditions explicit is the task of the researcher.

To obtain our results, we use a general causal inference method for eliciting and contrasting outcomes under hypothetical exposures in observational settings (e.g. Ahern et al., 2009; Naimi et al., 2017; Robins, 1986; Snowden et al., 2011). This method – variously referred to as *g-formula*, *g-computation* and *standardisation* (see Bendixen, 2023; Hernan & Robins 2020, chap. 13; VanderWeele et al. 2016) – involves three main steps: first, given a well-defined estimand and explicit causal assumptions, an appropriate statistical model is fitted that adjusts for relevant confounding variables. In the context of the present study, this first step is undertaken in Sections 3.2 and 4.3 below. Second, using the fitted model in the first step, we predict (or impute) outcome values under varying hypothetical levels of a focal predictor while holding all other variables as observed. This step follows directly from the interventionist approach to causal inference outlined above, in that it simulates changes in potential outcomes under manipulated, counter-factual exposures. Third, the imputed or predicted values from the previous step are contrasted and summarised by some appropriate inferential statistic, such as the mean and an interval. This step effectively amounts to averaging over the joint distribution of the observed covariates, yielding an *average* or *marginal* effect of the exposure in the sample. For the present analysis, for each individual and outcome response option, we compute the posterior mean under hypothetical exposure levels. For this effect to have a valid causal interpretation, a set of identifiability assumptions needs to be met (Hernan & Robins, 2020). We discuss these in Section 7 of the Supporting Information.

### 3.2. Causal model

**Figure 1.**
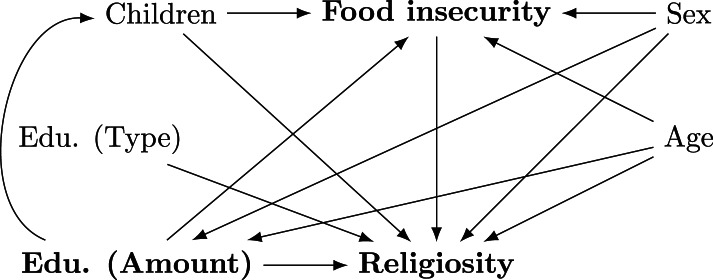
Directed acyclic graph of the assumed causal structure. Bold variables are focal variables.

Our guiding causal model (Figure 1) incorporates the hypotheses examined in the aforementioned literature (Section 2.2). The variables and causal relationships we consider are drawn from predicted directions of previous studies. The model is also informed and constrained by our knowledge of what variables we could use in the dataset.

The focal causal hypotheses are that religiosity increases as a function of exposure to education and food insecurity. To the extent that years of formal education constitute exposure to a particular form of instruction that has some effect on the degree to which one is religious, and concern about access to food approximates material insecurity, we use these direct effects as our focal theoretical predictions and empirical estimands: Education (Amount) → Religiosity, and Food Insecurity → Religiosity. We also incorporate some of the other aforementioned individual-level predictors into our model, including sex, age, and number of children. The model wagers that they comprise a causal network of influence between amount of education and religiosity. Specifically, it posits an internal causal structure to these variables: Sex → Food Security ← Age. If Children ← Food Security, conditioning on children would not be necessary if Food Security were held constant (for an introduction to causal graph analysis, see Pearl et al., 2016). However, if Children → Food Security, this creates a collider bias with Food Security and Education (Amount). We therefore take this more conservative route and hold the number of children constant. We also posit that Education (Amount) → Children. While this creates an indirect path through Children, as we are conditioning on number of children already and there are no other posited confounds, we are avoiding this problem. Furthermore, the amount of education should contribute to one's food security which should, in turn, contribute to religiosity. Sex and age confound the focal causal path, and there is an indirect effect of education amount on religiosity that runs through food security.

It is important to note that the *type* of education might matter here. We recognise that individuals could have had religious education in some contexts whether by default or by choice, and that choice could be facilitated by food security, and so forth. In this model, we posit only that it has an effect on religiosity. This is a very important assumption for a couple of reasons. First, this is unmeasured in the dataset. According to this model structure, however, its absence is inconsequential to estimating the target effects; we have no need for conditioning on type of education. If we posit that it was caused by food security, not having measured it would be inconsequential to the target estimate as long as we hold food security constant (i.e. the indirect effect through education type would be blocked). However, if the type of education were posited to cause food security, this would both confound the effect through food security, but also open a confounding path between education amount through food security. With this important note in mind, we continue under the assumption that type of education only affects religiosity. In Section 3 in the Supporting Information, we walk through a simulation of this model that shows that we can recover target estimates with and without this particular variable.

## 4. Study

### 4.1. Participants

**Figure 2.**
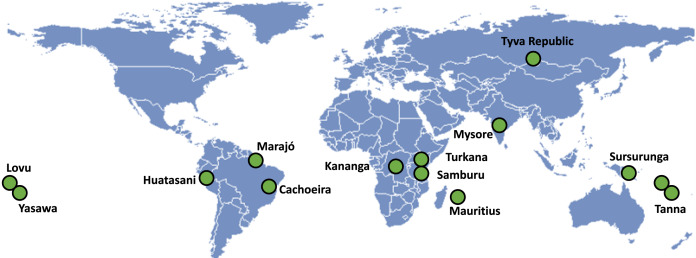
Map of 14 field sites. Note that there are two field sites in Tanna (Inland and Coastal).

We used data from the publicly available *Evolution of Religion and Morality Project* dataset (for motivation, design details, and further details about each field site, see Lang et al., 2019; Purzycki et al., 2016, 2018a, 2022b). The entire dataset consists of 2228 participants from 15 diverse populations around the world (see Fig. 2), but owing to there being no data on the outcome, we dropped one site (Hadza). Participants were recruited in a variety of ways; some of them were recruited on the basis of self-identification with a locally relevant religious tradition. There are no indications that recruitment on this basis contributed to the selection of individuals who are more religiously committed. All participants were 17 years of age or older. All participants engaged in a variety of economic game experiments designed to examine the breadth of religiously motivated cooperation. Participants were informed that their participation would be anonymous, any identifying information would be kept under lock and key and they had the right to withdraw at any point. Table 1 details the key descriptive statistics of each field site.

**Table 1.** Means (standard deviations) for some individual-level demographic variables and group-level properties of communities sampled. ‘Mjr trad.’ refers to the major colonial and/or overarching tradition of each site while ‘Mnr trad.’ refers to relatively minor – but locally salient – traditions in each site.

Culture	*N*	Males	Age	Years of education	Mjr trad.	Mnr trad.	Economy
Cachoeira	274	83	34.19 (12.87)	8.58 (4.02)	Christianity	Candomblé	Market
Coastal Tanna	178	88	35.14 (14.33)	7.76 (4.22)	Christianity	Traditional	Horticulture
Huatasani	94	37	38.51 (15.92)	8.96 (3.80)	Christianity	Traditional	Farming/Herding
Inland Tanna	112	57	36.25 (15.40)	0.68 (2.04)	Traditional	Traditional	Horticulture
Kananga	200	79	38.09 (14.46)	9.51 (3.32)	Christian	Traditional	Market
Lovu	76	24	44.56 (16.94)	8.77 (3.78)	Hindu	—	Market
Marajó	77	37	34.12 (13.08)	8.00 (3.53)	Christianity	Hagiolatry	Market
Mauritius	245	144	36.93 (15.80)	8.84 (3.57)	Hinduism	Sorcery	Market/Farming
Mysore	165	94	33.56 (12.34)	13.35 (5.42)	Hinduism	Hinduism	Market
Samburu	40	12	51.27 (12.48)	0.70 (1.76)	Christianity	Traditional	Herding
Sursurunga	163	73	37.60 (14.13)	7.51 (2.63)	Christianity	—	Horticulture
Turkana	247	91	38.03 (16.38)	0.48 (1.23)	Christianity	Traditional	Herding
Tyva Republic	81	23	33.53 (12.52)	15.44 (2.29)	Buddhism	Shamanism	Market/Herding
Yasawa	75	34	38.04 (15.91)	9.66 (2.42)	Christianity	Traditional	Fishing/Farming
Total	2027	876	36.82 (14.87)	7.68 (5.27)	—	—	—

### 4.2. Methods

In addition to our focal variables of formal education, food security and religiosity, we also include the ancillary demographic factors (sex, age and number of children). As some traditions emphasise and signal religious commitment variously through a combination of belief and practice (Baimel et al., 2022; Cohen et al., 2003), we consider two questions that represent religiosity or religious commitment. One focuses on intrinsic or ideational commitment: *How often do you think about* [*deity*]*?* The other focuses on extrinsic or behavioural commitment: *How often do you perform activities or practices to talk to or appease* [*deity*]*?* Response options were on a five-point frequency scale (1 = very rarely/never, 2 = a few times per year, 3 = a few times per month, 4 = a few times per week, 5 = every day or multiple times per day). We measured current food security with a binary, yes/no question (no = 0; yes = 1): *Do you worry that in the next month your household will have a time when it is not able to buy or produce enough food to eat?* (Hruschka et al., 2014).

### 4.3. Statistical model

To model the level of commitment across the two variables, we used Bayesian ordered-logistic regressions (Bürkner & Vuorre, 2019). In each population, *j*, we asked each individual, *i*, the commitment questions, outcomes of which, *K*_*i*_, are ordered categorical responses. As such, we model them using a multilevel ordered categorical likelihood distribution that is parameterised using a linear model term, *ξ*_*i*_, and a vector of random cut-points, *κ*:1
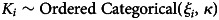


The linear model *ξ*_*i*_ is then given by:2
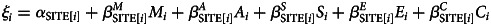
where SITE[*i*] gives the cultural group of individual *i*, *A*_*i*_ is the age of individual *i*, *S*_*i*_ is a variable indicating if individual *i* is male, *E*_*i*_ is the years of formal education completed by individual *i*, *C*_*i*_ indicates an individual's number of children and *M*_*i*_ indicates an individual's food insecurity. To account for the fact that increments in the predictor (e.g. an additional year of education and year of age or an additional child) might have differential associations with the outcome, we model the effects of years of education, age and number of children monotonically, such that a covariate's *β* coefficient represents the expected average difference between two adjacent levels of the predictor (Bürkner & Charpentier, 2020). Note that we also modelled age and years of education with Gaussian processes (see Section 4 in the Supporting Information) but found the monotonic model to yield a better fit. Food insecurity and sex are modelled as indicator variables. Since unobserved site-specific factors are likely to affect our predicted effects in different ways across sites, our statistical model fully varies effects across sites. It is plausible that, for example, one year of education in one place deeply affects one's worldview whereas in others, it might have no effect. By-site clustering partly handles site-specific confounds.

We define our priors, which we checked through prior predictive simulation (see Section 4 in the Supporting Information) as follows. The thresholds *κ* are modelled with Normal(0, 10), all *β* coefficients with Normal(0, 0.5) and all monotonic covariates with a Dirichlet distribution with *α* denoting a series of 2s equalling the number of levels of the predictor. The Dirichlet prior encodes the *a priori* assumption that any of the levels of the predictor could be more or less likely than the others (McElreath, 2020, pp. 392–394). Variance components for the varying effects were set at Exponential(1) and the correlation matrix of the variance components to LKJCorr(4) (Lewandowski et al., 2009). Taken together, the specified set of priors is weakly regularising.

We performed all analyses using the brms package (Bürkner, 2017, 2018, 2021) for R (R Core Team, 2021), an interface to the probabilistic programming language Stan (Carpenter et al., 2017). Chain and sampling diagnostics (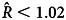, well-mixing trace and rank plots, few divergent transitions, and relatively large effective sample sizes) were acceptable. Section 8 in the Supporting Information includes the full list of R packages used for this project as well as their dependencies and version number.

### 4.4. Results

#### 4.4.1. Food insecurity

**Figure 3.**
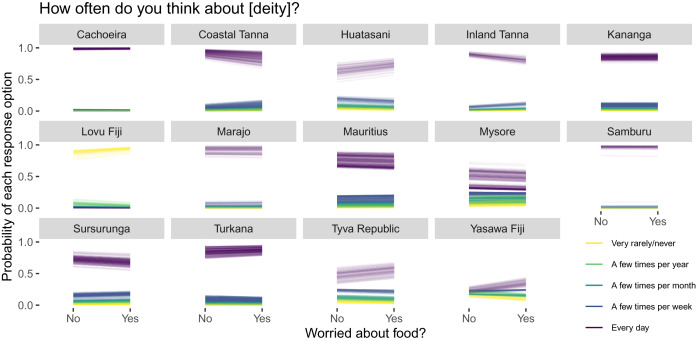
Food insecurity and religious ideation. Posterior means for each individual and outcome response option under hypothetical exposures to food (in)security.

**Figure 4.**
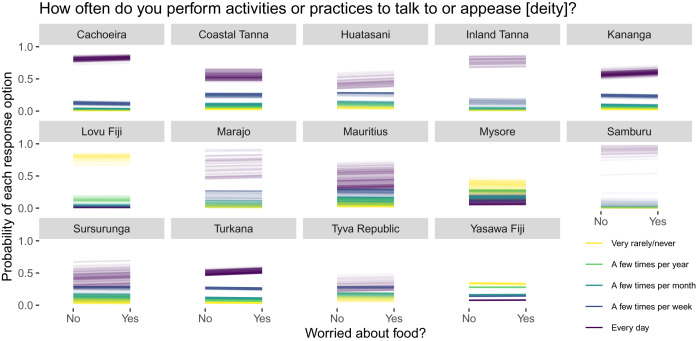
Food insecurity and religious behaviour. Posterior means for each individual and outcome response option under hypothetical exposures to food (in)security.

Figures 3 and 4 show predicted (posterior means) individual comparisons between reporting being worried about food and not being worried about food for each site and outcome response option. Consider that if food insecurity predicts religious commitment, we would expect that the higher outcome response options (i.e. thinking about and performing activities directed at the focal deity more often; the darker line colours) should become *more* probable (i.e. trend upwards), whereas the lower response options (i.e. thinking about and performing activities directed at the focal deity less often; the lighter line colours) should become *less* probable (i.e. trend downward) as a function of reporting food insecurity. Note that while we are plotting the functional relationship between food security and religious commitment linearly, this is for presentational purposes only; we statistically modelled food security as an indicator variable. While we see semblances of this pattern at a few sites (e.g. Figure 3 – Huatasani, Tyva Republic, Yasawa Fiji; Figure 4 – Kananga, Turkana), at other sites we see the opposite pattern (e.g. Figure 3: Coastal and Inland Tanna, Sursurunga) or, most commonly, no particular change in either direction.

#### 4.4.2. Education

**Figure 5.**
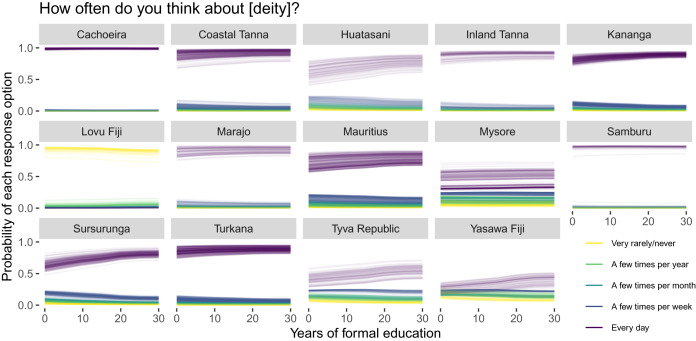
Years of formal education and religious ideation. Posterior means for each individual and outcome response option under hypothetical number of years of formal education.

**Figure 6.**
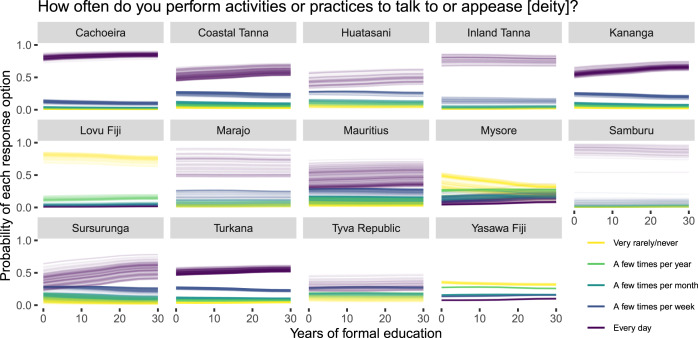
Years of formal education and religious behaviour. Posterior means for each individual and outcome response option under hypothetical number of years of formal education.

Figures 5 and 6 show predicted (posterior means) individual comparisons between a range of educational attainment levels for each site and outcome response option. Specifically, we obtained predictions for years of formal education from 0 to 30 years (the observed range in the sample) in increments of five. Here, if educational attainment negatively predicts religious commitment, we would expect that the higher outcome response options (i.e. thinking about and performing activities directed at the focal deity more often; the darker line colours) become *less* probable (i.e. trend downwards), whereas the lower response options (i.e. thinking about and performing activities directed at the focal deity less often; the lighter line colours) would become *more* probable (i.e. trend upwards) as a function of additional years of formal education. At most sites, we in fact find the opposite of this predicted pattern, such that additional years of formal education predict more religious commitment, although the associations generally seem small or moderate. At a few other sites, we do find the hypothesised pattern (e.g. Figure 5: Lovu Fiji; Figure 6: Lovu Fiji, Mysore), while at other sites no particular change is observed. Overall, there is no stable relationship.

## 5. Discussion

Overall and across sites, then, we find little-to-no reliable evidence that food security or educational attainment causes religious commitment. If anything, based on these data and our causal and statistical models, additional years of formal education tend to *increase* religious commitment at several sites, contrary to common theorising. We see the present work as taking some modest but necessary first steps in reliably estimating the causal relationship between educational attainment, food insecurity and religiosity specifically, and the material insecurity hypothesis more generally. We also hope this work can motivate a general workflow for cross-cultural causal research in the quantitative social sciences (cf. Bulbulia, 2022; Deffner et al., 2022). As we turn to now, there are some issues from theory and model to analysis, prediction, and inference to which future work should attend.

In terms of modelling, while our statistical model and analyses directly follow from our causal model, our causal model does not directly follow from a formal theoretical model. What theory we do have is informal and recognised as underdeveloped in a host of ways (Storm, 2017). Our approach gathered various hypotheses together, forged a synthetic causal model out of verbal theoretical synthesis and framed that theorising with an appeal to a set of formal cultural evolutionary models.

That said, as is often the case, the present study may be susceptible to biases from confounding variables our model did not consider. While it is unclear to us that recruitment was biased in terms of levels of religiosity, education, and the other factors we considered, our model is a necessary simplification of a complex process. Future efforts can build upon our model and consider further factors that may play an important role in estimating how education corresponds to religiosity. We anticipate that our causal model can function as foundation upon which to build. Our hypotheses are restricted to the posited direction of the effect of food security and education, but we have no precise sense of what the magnitude of the effect should be. More precision in theory would help here. It is also important to emphasise that the value of building and presenting a causal model, rather than attempting to get at one ‘true’ representation of the world, is to make one's assumptions transparent and explicit.

Indeed, making assumptions transparent and explicit is a critical step in causal inference, but it is only just a first step. As we showed using *g-computation*, calculating contrasts is a necessary follow-up to establish how outcomes fare in light of manipulating the focal predictor. Another next step is accounting for how generalisable results are. It is therefore important to stress that our main model and predictions yield inferences *for a particular population*, namely the sampled sites and participants with complete cases for the included variables, when hypothetically manipulating food security and years of formal education. While our dataset is both culturally and demographically diverse, our sample may not necessarily be characteristic of any given population for which one wishes to draw inference. For the sake of illustration, then, we also show how to go about obtaining predictions for a non-sampled but well-defined population using so-called *poststratification* (Deffner et al., 2022; Kennedy & Gelman, 2021) in Section 6 in the Supporting Information. Briefly put, poststratification involves re-weighting model-based predictions using weights obtained from external data that are more representative of a population of interest. Drawing from another data sample, we show how to use the model to predict religiosity in the context of a secular, materially secure and wealthy country (Denmark).

Other steps remain on the horizon. In particular, we anticipate improving inference using different sets of assumptions. In our case, had we found a notable target effect and the issue of education type (or any other missing confounder) was compelling enough, sensitivity analyses might detect how sizeable this unmeasured confound's effects would have to be to upend the estimated causal effect (Ding & VanderWeele, 2016; VanderWeele & Ding, 2017). Recent efforts (Major-Smith, 2023) bring a range of sensitivity analyses to bear on inferring causation in observational data of religious prosociality. Of course, more discussion and attention are necessary to determine what exactly qualifies as ‘compelling enough’ and what the criteria are by which we arrive at such conclusions.

Methodologically, more robust measures of food and other forms of insecurity would be useful to examine the range of well-being types corresponding to religiosity (for review of various methods, see Manikas et al., 2023). Alas, the dataset we used offers no such measures. More importantly perhaps is the specific aspects of formal education that might matter when it comes to playing a role in reducing (or increasing) religiosity. While our causal model suggests that we need not worry about the role of the type of education, there are other aspects of formal education that could play a clearer role in affecting religiosity. Rather than exposure to formal education in years, other individual-level factors such as performance, attendance and/or general enthusiasm in school might be pivotal for individual commitment to religion. Again, theory should help direct further inquiry.

More pragmatically, our results also have bearing on any potential interventions. For those committed to making higher education more available under the assumption that it will decrease commitment to religion, our results suggest that increasing the amount of education individuals obtain is not likely to do much. If our assumptions are realistic, the model is a worthy candidate for explanation and our data are representative of the kinds of variation we expect to see in the world, we can conclude that there are no general or reliable effects of education and food security on religiosity. It may be the case that educational attainment might lead to the kinds of social living that rely less on (or have less time for) religion and its benefits (Inglehart, 2020). Our model certaintly oversimplifies this process, but takes the first steps towards formally modelling it in a causal framework.

In closing, we hope this report usefully provides a template for future work causally investigating the material security hypothesis with either cross-sectional or longitudinal data. With the goal of making causal assumptions, models and use of theory explicit, we identified areas for future research and outlined a workflow for facilitating inference-making beyond what simple descriptions of data offer.

## Supporting information

Purzycki and Bendixen supplementary material 1 Purzycki and Bendixen supplementary material

Purzycki and Bendixen supplementary material 2 Purzycki and Bendixen supplementary material

Purzycki and Bendixen supplementary material 3 Purzycki and Bendixen supplementary material

Purzycki and Bendixen supplementary material 4 Purzycki and Bendixen supplementary material

## Data Availability

The data, code and supplementary materials for this particular study are hosted at https://github.com/tbendixen/causal-inference-schooling. The greater project from which the present work drew is hosted at https://github.com/bgpurzycki/Evolution-of-Religion-and-Morality.

## Supplementary material

The supplementary material for this article can be found at https://doi.org/10.1017/ehs.2023.29
