# Supplementary material for: Material insecurity and religiosity: A causal analysis
Source: Evol Hum Sci. 2024 Jan 17;6:e4. doi: 10.1017/ehs.2023.29 (PMC10955381; doi:10.1017/ehs.2023.29)
Supplement: Purzycki and Bendixen supplementary material 4 — Purzycki and Bendixen supplementary material [file S2513843X23000294sup004.pdf]

Material insecurity and religiosity: A causal analysis

Benjamin Grant Purzycki Theiss Bendixen

11/1/23

Table of contents

1 Introduction **2**
1.1 Set-up and data preparation . . . 2

2 Simpson's "paradox" **5**

3 Simulation **8**

4 Monotonic vs. Gaussian process **11**
4.1 Monotonic global model . . . 12
4.2 Gaussian process global model . . . 13
4.3 Monotonic multilevel model . . . 14
4.4 Gaussian process multilevel model . . . 15
4.5 Model comparison . . . 16
4.6 Final model . . . 17

5 Results **18**
5.1 Food security . . . 19
5.2 Years of formal education . . . 22

6 Multilevel Regression with Poststratification **25**

7 Identifiability assumptions **33**

8 R packages, versions, and dependencies **34**

9 References **36**

For the most recent version of the notebook, see: https://github.com/tbendixen/causal-inference-schooling

1 Introduction

This notebook fits and reports on the main and supplementary models for our analysis on religious commitment as predicted by food insecurity and educational attainment.

We first discuss and demonstrate the impact on inference between accounting versus not accounting for group-level structures – sometimes referred to as Simpson's "paradox".

Next, we discuss the causal simulation exercise mentioned in the main manuscript.

We then fit and compare two approaches to modeling years of formal education and age – namely, via Gaussian processes and monotonic effects.

Then, we fit our main analysis, as reported in the submitted manuscript and compare the marginal predictions to a model that post-stratify predictions to a different, non-sampled population. We also briefly discuss the key identifiability assumptions underlying our g-computation approach.

Finally, we list and cite all `R` packages, their dependencies, and version number used for this project.

1.1 Set-up and data preparation

We first load relevant packages and specify the path to `cmdstan`, the engine for fitting Bayesian models. We then read in the data and subset to relevant variables and complete cases. We then set the variables to their respective types. We also write generic fitting functions.

```
# Load packages
library(tidybayes) # for post-processing and plotting
library(brms) # for Bayesian modeling
library(dplyr) # for data preparation
library(ggplot2) # for plotting
library(grateful) # for citing packages
library(patchwork) # for panel plots

# Increase memory allocation
memory.limit(size=56000)
```

```
[1] Inf
```

```r
# Set path to whereever cmdstan in installed
cmdstanr::set_cmdstan_path("C:\\cmdstan\\cmdstan-2.29.0")

# Read data and retain only complete cases
d <- read.delim("ALLSITES_V3.7_tabdel.txt")

labs <- c("SITE", "CID", "AGE", "SEX", "CHILDREN",
          "FORMALED", "MAT1",
          "BGTHINK", "BGPERFHO")
d <- d[labs]
d <- d[complete.cases(d), ]

# Prepare for ordered categorical modeling
d$BGTHINK <- factor(d$BGTHINK, ordered = T)
d$BGPERFHO <- factor(d$BGPERFHO, ordered = T)

# binary variables as factors
d$SEX <- factor(d$SEX)
d$MAT1 <- factor(d$MAT1)

# Prepare monotonic modeling
d$FORMALED.mo <- factor(d$FORMALED, ordered = T)
d$AGE.mo <- factor(d$AGE, ordered = T)
d$CHILDREN.mo <- factor(d$CHILDREN, ordered = T)

# Generic fitting functions
fit_brms <- function(formula, priors, data) {
  brm(formula = formula,
      data = data,
      iter = 1000, cores = 4,
      prior = priors,
      family = cumulative("logit"),
      backend = "cmdstanr",
      seed = 1992)
}

# For prior predictive check
fit_brms_ppc <- function(formula, priors, data) {
  brm(formula = formula,
      data = data,
      iter = 1000, cores = 4,
```

```
      sample_prior = "only",
      prior = priors,
      family = cumulative("logit"),
      backend = "cmdstanr",
      seed = 1992)
}

# For simple prior vs. posterior check
prior_vs_post <- function(prior_model, post_model) {
  ndraws <- 100
  set.seed(1992)

pvp <- (pp_check(prior_model,
            resp = "BGTHINK",
            ndraws = ndraws,
            type = "bars",
            size = 0.1,
            freq = FALSE)) +
  theme(legend.position = "none") +
  labs(title = "Prior predictive check",
       subtitle = "How often do you think about [deity]?") +
  ylim(c(0,1)) +
      (pp_check(prior_model,
              resp = "BGPERFHO",
              ndraws = ndraws,
              type = "bars",
              size = 0.1,
              freq = FALSE)) +
     theme(legend.position = "none") +
     labs(title = "Prior predictive check",
          subtitle = "How often do you perform activities or practices\n
           ↪  to talk to or appease [deity]?") +
     ylab(NULL) +
     ylim(c(0,1)) +
          (pp_check(post_model,
                  resp = "BGTHINK",
                  ndraws = ndraws,
                  type = "bars",
                  size = 0.1,
                  freq = FALSE)) +
        theme(legend.position = "none") +
```

```
          labs(title = "Posterior predictive check",
          subtitle = "How often do you think about [deity]?") +
         ylim(c(0,1)) +
             (pp_check(post_model,
                   resp = "BGPERFHO",
                   ndraws = ndraws,
                   type = "bars",
                   size = 0.1,
                   freq = FALSE)) +
           theme(legend.position = "none") +
           labs(title = "Posterior predictive check",
           subtitle = "How often do you perform activities or practices\n
            ↪  to talk to or appease [deity]?") +
           ylab(NULL) +
           ylim(c(0,1)) +
               patchwork::plot_layout(ncol = 2, nrow = 2)

  set.seed(NULL)

  return(pvp)
}
```

2 Simpson's "paradox"

As reviewed in the main manuscript, some previous research analyzes group-level association between various sociodemographic factors and religious commitment and finds, for instance, that years of education negatively predicts religiosity. Here, using simple bivariate regressions, we show how analyzing group-mean aggregated can potentially lead us astray. This is sometimes referred to as Simpson's paradox, although technically there's no paradox. It's simply a result of nested data structures.

Specifically, in the following graphs, we replicate previous findings that, on a group-mean aggregate level, there's a negative relationship between education and religiosity (left panel), but that this relationship disappears when taking into account the grouping structure in the data (namely, the field sites; right panel). One way to think of this phenomenon is to consider the field sites as proxies for unobserved, site-specific confounding factors that are (at least partly) accounted for, when allowing each site to have its own intercept and slope.

```
d_agg <- with(d, data.frame(
  SITE = unique(d$SITE),
```

```
  FORMALED = aggregate(as.numeric(FORMALED), list(SITE), FUN = mean)$x,
  BGTHINK = aggregate(as.numeric(BGTHINK), list(SITE), FUN = mean)$x,
  BGPERFHO = aggregate(as.numeric(BGPERFHO), list(SITE), FUN = mean)$x)
  )

(d_agg |> ggplot(aes(x = FORMALED, y = BGTHINK)) +
  geom_smooth(method = "lm", se = T) +
  geom_jitter(size = 2) +
  theme(panel.grid.minor = element_blank(),
        panel.background = element_rect(fill="white")) +
  scale_y_continuous(breaks=c(1,2,3,4,5)) +
  xlab("Years of formal education (group-mean)") +
  ylab("Religious ideation (group-mean)") +
  labs(title = "Simpson's ''paradox''",
       subtitle = "Formal education and religious ideation")) +
(d |> ggplot(aes(x = as.numeric(FORMALED), y = as.numeric(BGTHINK),
 ↪  color = SITE)) +
  geom_jitter(alpha=0.05) +
  facet_wrap(~ SITE) +
  geom_smooth(method = "lm", se = T) +
  theme(legend.position = "none",
        panel.grid.minor = element_blank(),
        panel.background = element_rect(fill="white")) +
  scale_y_continuous(breaks=c(1,2,3,4,5)) +
  xlab("Years of formal education") +
  ylab("Religious ideation")) +
  plot_layout(ncol=2)
```

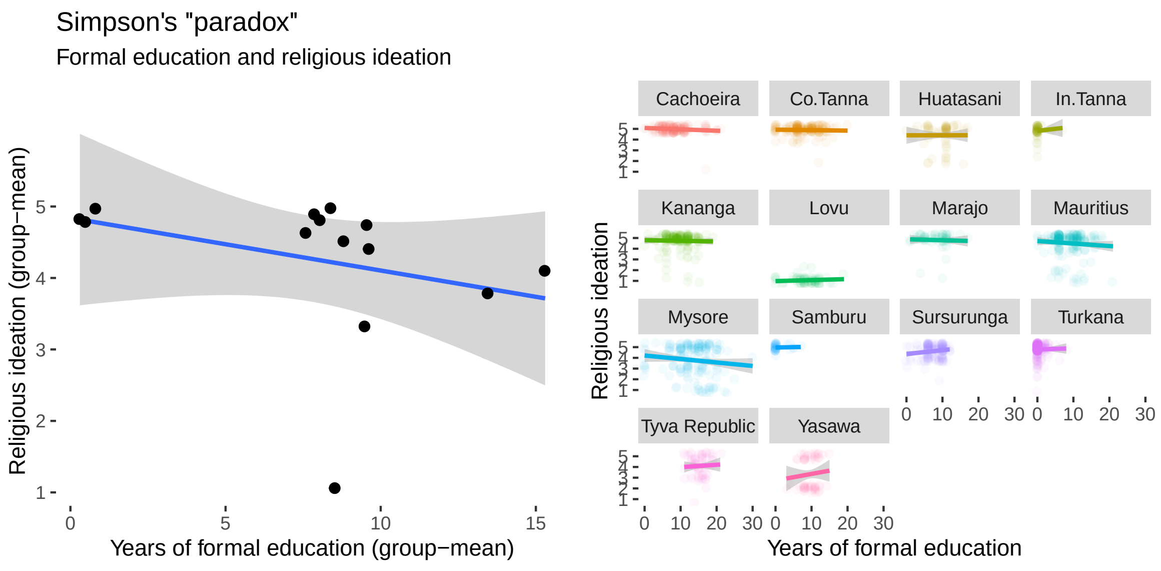


```r
(d_agg |> ggplot(aes(x = FORMALED, y = BGPERFHO))) +
  geom_smooth(method = "lm", se = T) +
  geom_jitter(size = 2) +
  theme(panel.grid.minor = element_blank(),
        panel.background = element_rect(fill="white")) +
  scale_y_continuous(breaks=c(1,2,3,4,5)) +
  xlab("Years of formal education (group mean)") +
  ylab("Religious behavior (group mean)") +
  labs(title = "Simpson's ''paradox''",
       subtitle = "Formal education and religious behavior") +
(d |> ggplot(aes(x = as.numeric(FORMALED), y = as.numeric(BGPERFHO),
↪   color = SITE))) +
  geom_jitter(alpha=0.05) +
  facet_wrap(~ SITE) +
  geom_smooth(method = "lm", se = T) +
  theme(legend.position = "none",
        panel.grid.minor = element_blank(),
        panel.background = element_rect(fill="white")) +
  scale_y_continuous(breaks=c(1,2,3,4,5)) +
  xlab("Years of formal education") +
  ylab("Religious behavior") +
  plot_layout(ncol=2)
```

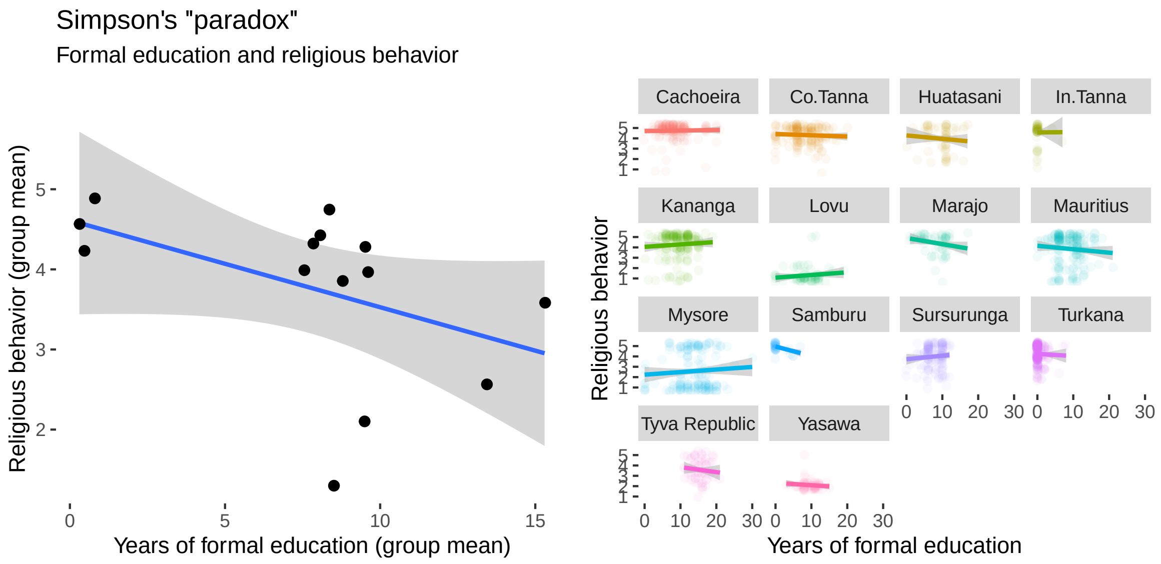

3 Simulation

Recall our DAG from the main text.

```
knitr::include_graphics("DAG-1.png")
```

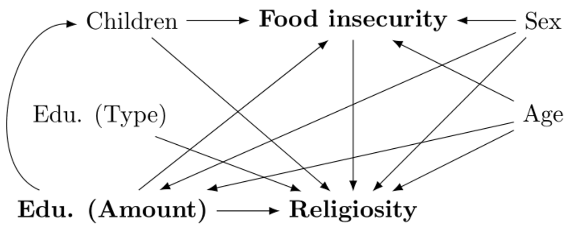

To ensure that this model was resolvable, we simulated it, treating sex and education type as binary variables, each with a 50% chance of having a value of 1. The remaining variables $\sim$ Normal$(0, 1)$, including the error terms we added to each variable for some realistic noise.

For the sake of simplicity, we hard-coded each causal estimand at 0.5. Results are distributions of estimates from 1,000iterations where a single run sampled from 1,000 individuals. As illustrated in the following figure, we recovered this value when we condition on sex, food security and age. And, as suggested by the causal graph, holding education type constant is inconsequential.

```r
# Simulation of DAG
colorshex <- c("#c9b2c8", "#070808", "#6c90a1", "#55758c", "#FF0000") #
↪  Stoner Witch palette
colorsrgb <- col2rgb(colorshex)

mycol1 <- rgb(201, 178, 200, max = 255, alpha = 200, names = "Lily")
mycol2 <- rgb(7, 8, 8, max = 200, alpha = 200, names = "Woodsmoke")
mycol3 <- rgb(108, 144, 161, max = 200, alpha = 200, names = "Gothic")
mycol4 <- rgb(85, 117, 140, max = 200, alpha = 200, names = "Smalt
↪  Blue")
mycol5 <- rgb(255, 0, 0, max = 300, alpha = 200, names = "red")

fd <- function(n, beta) {
  e_rel <- rnorm(n, 0, 1) # noise
  e_edu <- rnorm(n, 0, 1)
  e_mat <- rnorm(n, 0, 1)
  e_qua <- rnorm(n, 0, 1)
  e_sex <- rnorm(n, 0, 1)
  e_age <- rnorm(n, 0, 1)
  e_kid <- rnorm(n, 0, 1)
  SEX <- rbinom(n, 1, .5) # sex
  XIA <- rbinom(n, 1, .5) # edu type
  PRI <- rbinom(n, 1, .5) # prime
  AGE <- rnorm(n, 0, 1) # age
  EDU <- beta * SEX + beta * AGE + e_edu # edu
  MAT <- beta * SEX * beta * EDU + beta * AGE + e_mat # food security
  KID <- beta * MAT + beta * AGE + + beta * EDU + e_kid # children
  REL <- beta * EDU + beta * XIA + beta * MAT + beta * SEX + beta * AGE
↪  +
    beta * PRI + beta * KID + e_rel # religiosity
  df <- data.frame(SEX, XIA, PRI, AGE, EDU, MAT, REL)
  open0 <- coef(lm(REL ~ EDU, dat = df))[2]
  open1 <- coef(lm(REL ~ EDU + SEX, dat = df))[2]
  open2 <- coef(lm(REL ~ EDU + SEX + MAT, dat = df))[2]
  open3 <- coef(lm(REL ~ EDU + SEX + MAT + AGE + KID, dat = df))[2]
  closed <- coef(lm(REL ~ EDU + SEX + MAT + AGE + + KID + XIA, dat =
↪  df))[2]
  withprime <- coef(lm(REL ~ EDU + SEX + MAT + AGE + + KID + XIA + PRI,
↪  dat = df))[2]
  return(c(open0, open1, open2, open3, closed, withprime))
}
```

```
sim1 <- data.frame(t(replicate(1000, fd(1000, .5))))
names(sim1) <- c("open0", "open1", "open2", "open3", "controlled",
 ↪  "withprime")

densop0 <- density(sim1$open0)
densop1 <- density(sim1$open1)
densop2 <- density(sim1$open2)
densop3 <- density(sim1$open3)
densco1 <- density(sim1$controlled)
#denspr1 <- density(sim1$withprime)

par(mar = c(3, 1, 1, 1))
plot(NA, xlab = NA, ylab = "",
     xlim = c(0.3, 1.5),
     ylim = c(0, 13),
     cex.lab = 1.3, yaxt = 'n')
polygon(densop0, col = mycol1) # open0
polygon(densop1, col = mycol2) # open1
polygon(densop2, col = mycol3) # open2
polygon(densop3, col = mycol4) # open3
polygon(densco1, col = mycol5) # closed
#polygon(denspr1, col = mycol1) # with prime
abline(v = 0.5, lty = 2)
legend(0.6, 12, legend = c("~ Edu. (Amnt.)", "+ Sex", "+ Mat. Sec.", "+
 ↪  Age + Kids",
                          "+ Edu (Type)"),
       fill = c(mycol1, mycol2, mycol3, mycol4, mycol5),
       cex = .7, horiz = F, bty = T, inset = c(0.03, 0.15))
```

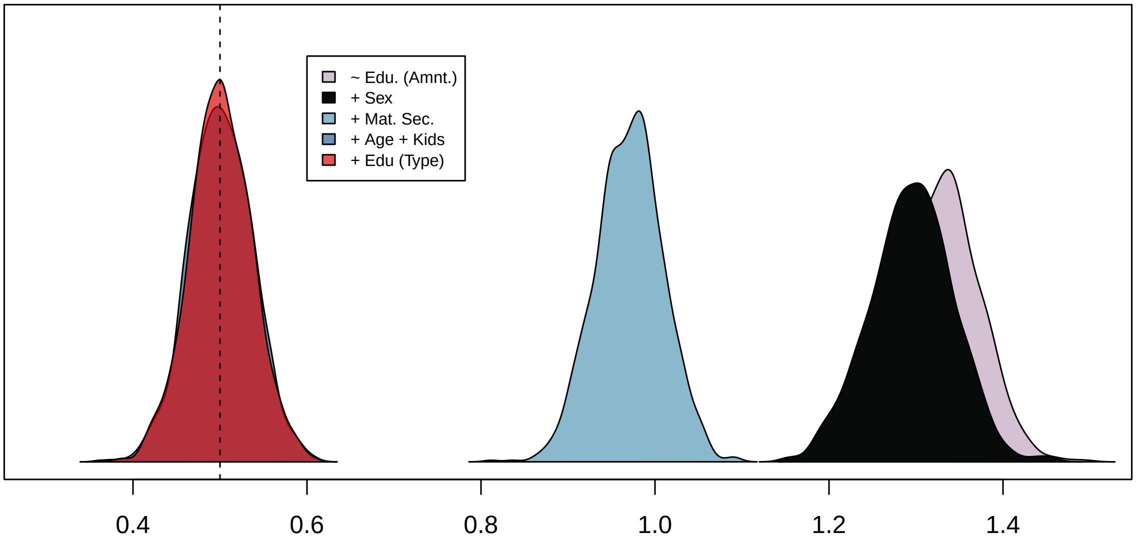

One important point about the data set is important to note in light of our causal model. Some participants included were part of experiments in the proximity of a religious prime (e.g., participating near a Bible, Buddhist charm, etc.). While these primes had little to no discernible effects on experiments, it might be nevertheless construed as as an important variable that may have increased reported religiosity during the data collection process. If we therefore updated the DAG above and included prime as a causal factor in religiosity, it would be inconsequential to condition on it because, like Education (Type), it poses no confounding influence on education amount and religiosity (or any other variable). We therefore did not include it in our analyses. The supplementary code nevertheless includes these factors for further exploration.

4 Monotonic vs. Gaussian process

In this section, we fit four models. One set of models approach years of formal education and age as monotonic, first without (`m1mo`) and then with (`m2mo`) by-site clustering.

The second set of models handles years of formal education and age with Gaussian processes, again without (`m1gp`) and with (`m2gp`) by-site clustering. We then compare the models (`m1mo` vs. `m1gp` vs. `m2mo` vs. `m2gp`), using approximate leave-one-out cross-validation (Vehtari, Gelman, and Gabry 2017; Yao et al. 2017; **loo2020a?**).

For the monotonic models, we specify mildly regularizing priors on all parameters. For the Gaussian process models, we specify mildly regularizing priors on the cut-points (what `brms` refers to as "Intercepts") but stick with the default priors for the Gaussian process parameters

themselves. Priors potentially play an important role in Gaussian process models and `brms` defaults to priors that are mildly catered to the data at hand (without encoding any particular relationship *a priori*).

In any case, our dataset is fairly large and to ensure that the posterior is in fact informed by the data, we plot convenient prior vs. posterior predictive checks for each model in turn. For computational ease, the prior vs. posterior checks are based on 100 draws from the posterior, so the point estimates (posterior medians) and 90% intervals are only indicative of the full posterior distribution.

4.1 Monotonic global model

```
m1mopriors <- set_prior("normal(0,0.5)",
                        class = "b",
                        resp = "BGTHINK") +
              set_prior("normal(0,0.5)",
                        class = "b",
                        resp = "BGPERFHO") +
              set_prior("normal(0,10)",
                        class = "Intercept",
                        resp = "BGTHINK") +
              set_prior("normal(0,10)",
                        class = "Intercept",
                        resp = "BGPERFHO") +
              set_prior("dirichlet(2)",
                        class = "simo",
                        resp = "BGTHINK",
                        coef = "moAGE.mo1") +
              set_prior("dirichlet(2)",
                        class = "simo",
                        resp = "BGTHINK",
                        coef = "moFORMALED.mo1") +
              set_prior("dirichlet(2)",
                        class = "simo",
                        resp = "BGPERFHO",
                        coef = "moAGE.mo1") +
              set_prior("dirichlet(2)",
                        class = "simo",
                        resp = "BGPERFHO",
                        coef = "moFORMALED.mo1")
```

```
m1moformula <- mvbind(BGTHINK, BGPERFHO) ~ mo(FORMALED.mo) + mo(AGE.mo)

m1moppc <- fit_brms_ppc(formula = m1moformula, priors = m1mopriors, data
↪ = d)

m1mo <- fit_brms(formula = m1moformula, priors = m1mopriors, data = d)
```

```
prior_vs_post(m1moppc, m1mo)
```

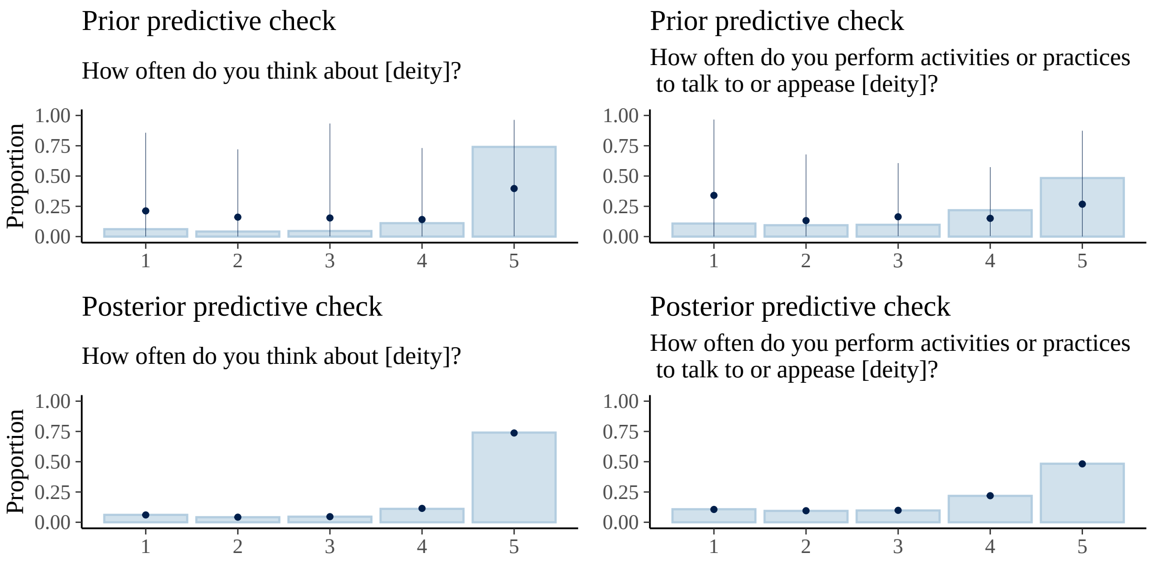

4.2 Gaussian process global model

```
m1gppriors <- set_prior("normal(0,2.5)",
                        class = "Intercept",
                        resp = "BGTHINK") +
              set_prior("normal(0,2.5)",
                        class = "Intercept",
                        resp = "BGPERFHO")

m1gpformula <- mvbind(BGTHINK, BGPERFHO) ~ gp(FORMALED) + gp(AGE)
```

```
m1gpppc <- fit_brms_ppc(formula = m1gpformula, priors = m1gppriors, data
  = d)

m1gp <- fit_brms(formula = m1gpformula, priors = m1gppriors, data = d)
```

```
prior_vs_post(m1gpppc, m1gp)
```

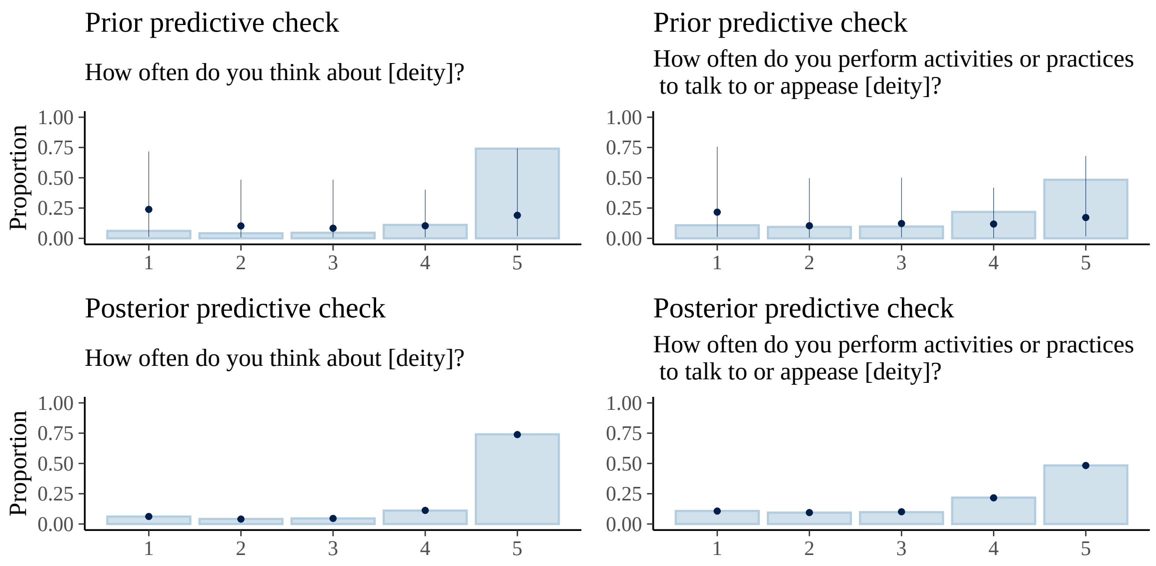

4.3 Monotonic multilevel model

```
m2mopriors <- m1mopriors +
              set_prior("lkj_corr_cholesky(4)",
                        class = "L")

m2moformula <- mvbind(BGTHINK, BGPERFHO) ~ mo(FORMALED.mo) + mo(AGE.mo)
  + (mo(FORMALED.mo) + mo(AGE.mo) | SITE)

m2moppc <- fit_brms_ppc(formula = m2moformula, priors = m2mopriors, data
  = d)

m2mo <- fit_brms(formula = m2moformula, priors = m2mopriors, data = d)
```

```r
prior_vs_post(m2moppc, m2mo)
```

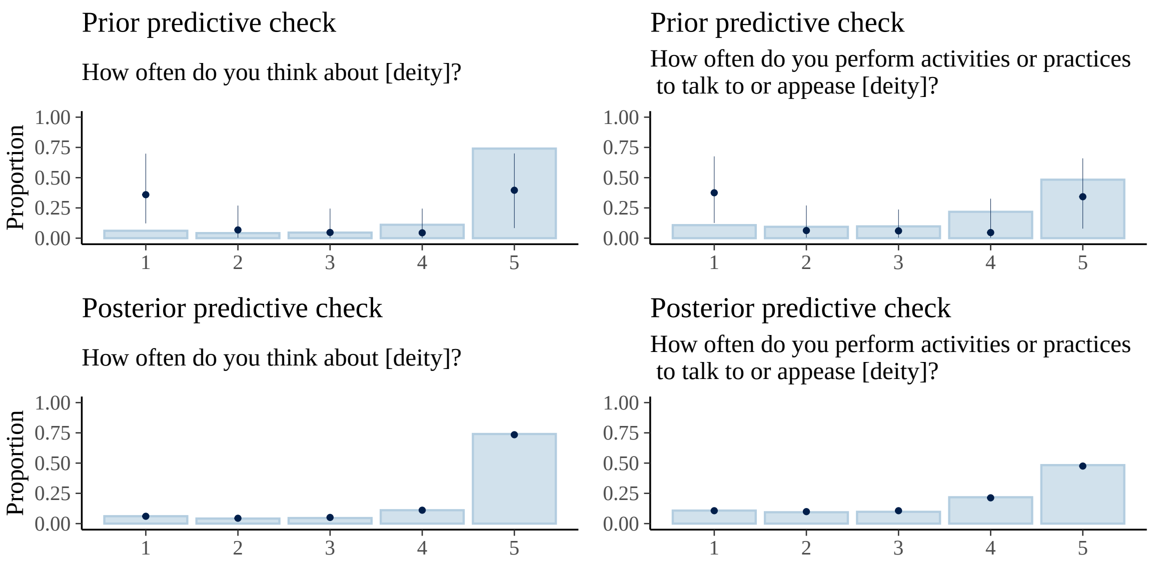

4.4 Gaussian process multilevel model

```r
m2gpformula <- mvbind(BGTHINK, BGPERFHO) ~ gp(FORMALED, by = SITE) +
  ↪ gp(AGE, by = SITE)

m2gpppc <- fit_brms_ppc(formula = m2gpformula, priors = m1gppriors, data
  ↪ = d)

m2gp <- fit_brms(formula = m2gpformula, priors = m1gppriors, data = d)
```

```r
prior_vs_post(m2gpppc, m2gp)
```

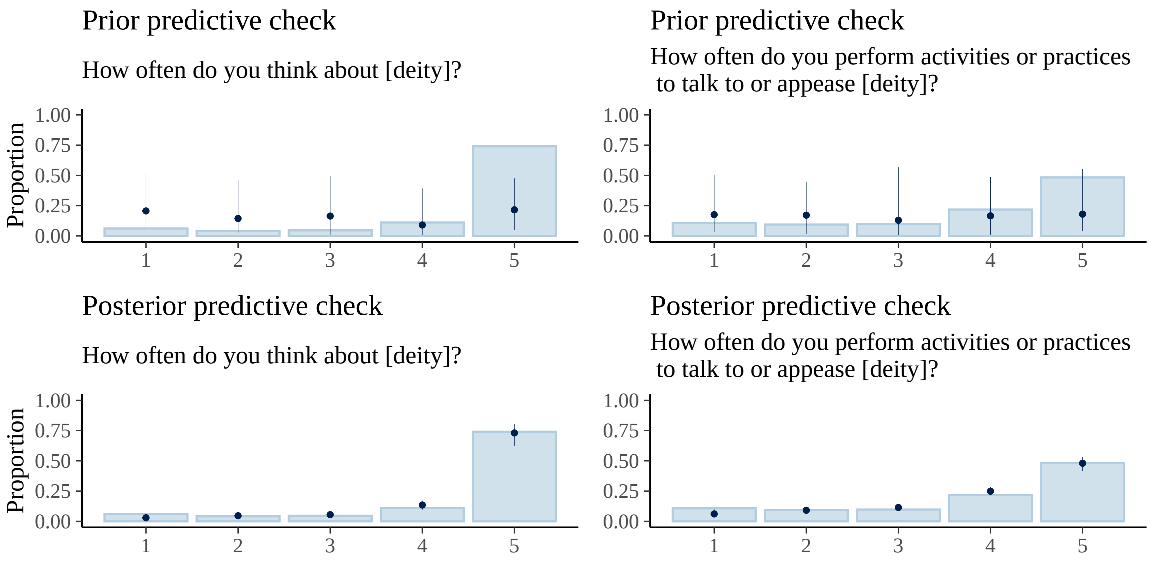

4.5 Model comparison

Comparing `m1mo` vs. `m1gp` vs. `m2mo` vs. `m2gp`:

```
loo_result <- loo::loo_compare(loo(m1mo), loo(m2mo), loo(m1gp),
    loo(m2gp))

loo_result
```

```
     elpd_diff se_diff
m2mo     0.0       0.0
m1gp  -627.4      46.4
m1mo  -628.2      46.6
m2gp -1463.5      90.7
```

Note that for the multilevel Gaussian process, we had some mild convergence issues, which are likely to also impact the model comparison results. We suspect that some of these issues could be solved using a combination of longer chains, stronger priors, and/or informative scaling and centering of the independent variables.

However, given that the monotonic effects models perform just as well or even better than the Gaussian process models and are also arguably more parsimonious in that we do not have to assume any particular density kernel *a priori* (as with the Gaussian process model), we proceed with the monotonic effects parameterization.

4.6 Final model

```
m3mopriors <- set_prior("normal(0,0.5)", class = "b", resp = "BGTHINK")
 ↪  +
              set_prior("normal(0,0.5)", class = "b", resp = "BGPERFHO")
               ↪  +
              set_prior("normal(0,10)", class = "Intercept", resp =
               ↪  "BGTHINK") +
              set_prior("normal(0,10)", class = "Intercept", resp =
               ↪  "BGPERFHO") +
              set_prior("exponential(1)", class = "sd", resp =
               ↪  "BGTHINK") +
              set_prior("exponential(1)", class = "sd", resp =
               ↪  "BGPERFHO") +
              set_prior("dirichlet(2)", class="simo", resp = "BGTHINK",
               ↪  coef = "moAGE.mo1") +
              set_prior("dirichlet(2)", class="simo", resp = "BGTHINK",
               ↪  coef = "moCHILDREN.mo1") +
              set_prior("dirichlet(2)", class="simo", resp = "BGTHINK",
               ↪  coef = "moFORMALED.mo1") +
              set_prior("dirichlet(2)", class="simo", resp = "BGPERFHO",
               ↪  coef = "moAGE.mo1") +
              set_prior("dirichlet(2)", class="simo", resp = "BGPERFHO",
               ↪  coef = "moCHILDREN.mo1") +
              set_prior("dirichlet(2)", class="simo", resp = "BGPERFHO",
               ↪  coef = "moFORMALED.mo1") +
              set_prior("lkj_corr_cholesky(4)", class = "L")

m3moformula <- mvbind(BGTHINK, BGPERFHO) ~ mo(FORMALED.mo) + mo(AGE.mo)
 ↪  + SEX + MAT1 + mo(CHILDREN.mo) +
                    (mo(FORMALED.mo) + mo(AGE.mo) + SEX + MAT1 +
 ↪  mo(CHILDREN.mo) | SITE)

m3moppc <- fit_brms_ppc(formula = m3moformula, priors = m3mopriors, data
 ↪  = d)

m3mo <- fit_brms(formula = m3moformula, priors = m3mopriors, data = d)
```

```
prior_vs_post(m3moppc, m3mo)
```

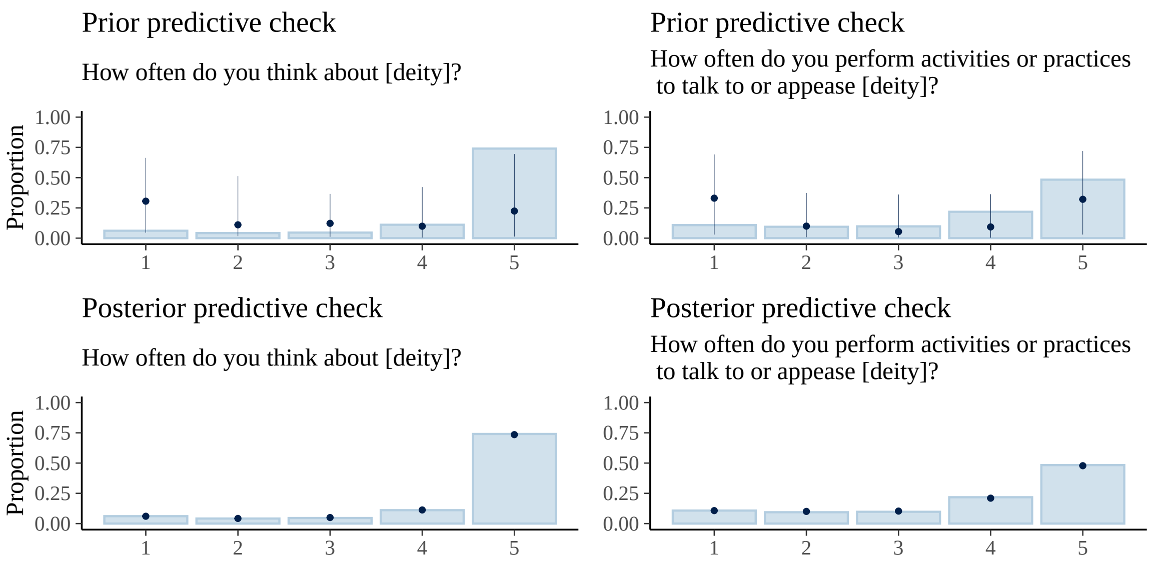

We run model comparison to check if the final model indeed performs predictively better than the more simple models. Results indicate that this indeed is the case, although the difference between `m3mo` and `m2mo` is relatively small compared to its standard error.

```
loo_result_fin <- loo::loo_compare(loo(m1mo), loo(m2mo), loo(m1gp),
↪  loo(m2gp), loo(m3mo))

loo_result_fin
```

```
     elpd_diff se_diff
m3mo      0.0       0.0
m2mo     -3.8       6.1
m1gp   -631.2      46.3
m1mo   -632.0      46.5
m2gp -1467.3      90.8
```

5 Results

Here, we produce the result plots reported in the main manuscript.

5.1 Food security

```
g_comp_bin <- function(model, resp) {

  set.seed(1992)

  # prediction grid
  predgrid <- rbind(transform(d, MAT1 = 0),
                    transform(d, MAT1 = 1))

  # get fitted values
  epred <- add_epred_draws(object = model,
                           ndraws = 2000,
                           newdata = predgrid,
                           re_formula = NULL,
                           resp = resp) |>
    mutate(category = factor(as.numeric(.category)))

  set.seed(NULL)

  # compute posterior means for ID and response option
  fitted <- epred |>
    group_by(CID, MAT1, SITE, category) |>
    summarize(post_mean = mean(.epred)) |>

    # prepare line plot
    group_by(CID, category) |>
    mutate(indices = cur_group_id()) |>
    ungroup()

  # prepare facet labels
  site_labels <- c("Co.Tanna" = "Coastal Tanna",
                   "In.Tanna" = "Inland Tanna",
                   "Sursurunga" = "Sursurunga",
                   "Turkana" = "Turkana",
                   "Samburu" = "Samburu",
                   "Huatasani" = "Huatasani",
                   "Mysore" = "Mysore",
                   "Cachoeira" = "Cachoeira",
                   "Kananga" = "Kananga",
                   "Mauritius" = "Mauritius",
```

```r
                    "Lovu" = "Lovu Fiji",
                    "Marajo" = "Marajo",
                    "Tyva Republic" = "Tyva Republic",
                    "Yasawa" = "Yasawa Fiji")

  # plot
  plot <- fitted |>
      ggplot(aes(x = MAT1,
                 y = post_mean,
                 group = indices,
                 color = category))  +
      geom_line(alpha = 0.05) +
      viridis::scale_color_viridis(discrete = TRUE,
                                   option = "D",
                                   name = NULL,
                                   direction = -1,
                                   labels=c("Very rarely/never",
                                            "A few times per year",
                                            "A few times per month",
                                            "A few times per week",
                                            "Every day")) +
    guides(colour = guide_legend(override.aes = list(alpha = 1))) + #
    ↪  controls the legend alpha separately from the geom_line() alpha
    facet_wrap(~SITE, nrow = 3, ncol = 5, labeller =
    ↪  as_labeller(site_labels)) +
    scale_x_continuous(name="Worried about food?", limits = c(-0.5,1.5),
    ↪  breaks = c(0,1), labels=c("No","Yes") ) +
    scale_y_continuous(name="Probability of each response option",
    ↪  breaks=c(0, .5, 1)) +
    theme(legend.position = c(0.92, 0.1),
          legend.text=element_text(size=7),
          legend.key=element_blank(),
          panel.grid.minor = element_blank(),
          panel.background = element_rect(fill="white"),
          legend.background = element_rect(fill="transparent"))

  set.seed(NULL)

  return(plot)
}
```

```
g_comp_bin(m3mo, "BGTHINK") + ggtitle("How often do you think about
  [deity]?")
```

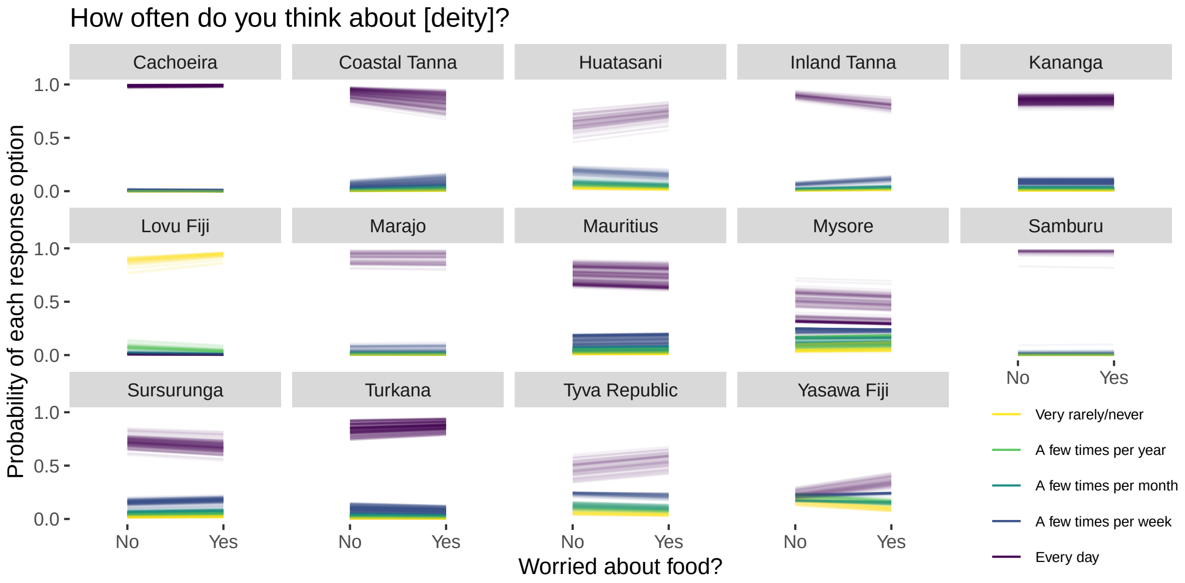


```
g_comp_bin(m3mo, "BGPERFHO") + ggtitle("How often do you perform
  activities or practices to talk to or appease [deity]?")
```

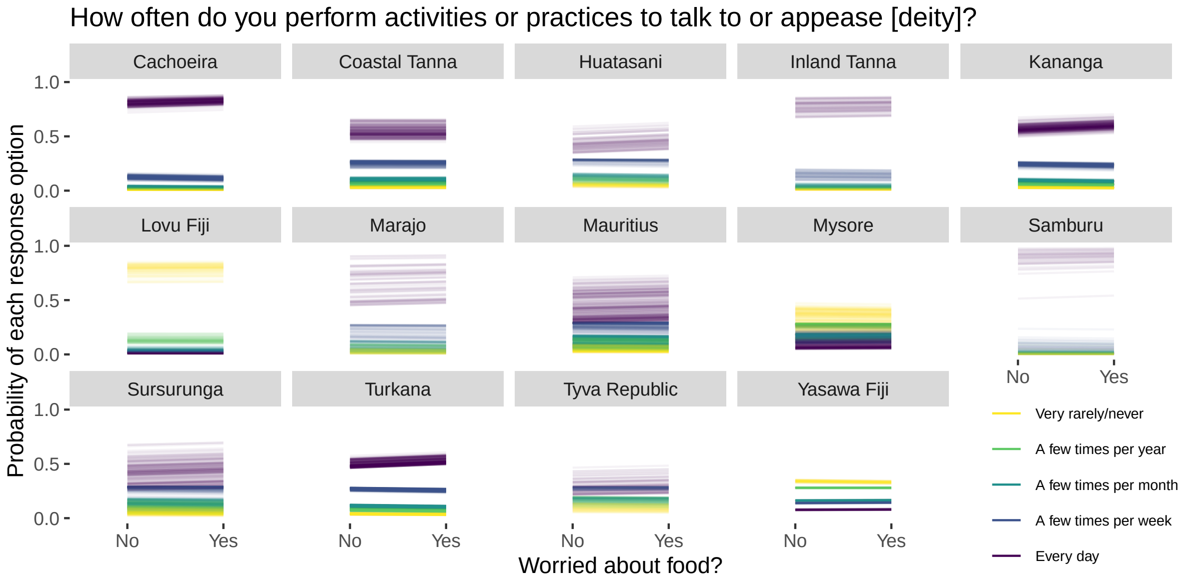

5.2 Years of formal education

```
g_comp_trend <- function(model, resp) {

  set.seed(1992)

  # prediction grid
  predgrid <- rbind(transform(d, FORMALED.mo = 0),
                    transform(d, FORMALED.mo = 5),
                    transform(d, FORMALED.mo = 10),
                    transform(d, FORMALED.mo = 15),
                    transform(d, FORMALED.mo = 20),
                    transform(d, FORMALED.mo = 25),
                    transform(d, FORMALED.mo = 30))

  # get fitted values
  epred <- add_epred_draws(object = model,
                           ndraws = 2000,
                           newdata = predgrid,
                           re_formula = NULL,
                           resp = resp) |>
    mutate(category = factor(as.numeric(.category)))

  set.seed(NULL)

  # compute posterior means for ID and response option
  fitted <- epred |>
    group_by(CID, FORMALED.mo, SITE, category) |>
    summarize(post_mean = mean(.epred)) |>

    # prepare line plot
    group_by(CID, category) |>
    mutate(indices = cur_group_id()) |>
    ungroup()

  # prepare facet labels
  site_labels <- c("Co.Tanna" = "Coastal Tanna",
                   "In.Tanna" = "Inland Tanna",
                   "Sursurunga" = "Sursurunga",
                   "Turkana" = "Turkana",
                   "Samburu" = "Samburu",
```

```
                    "Huatasani" = "Huatasani",
                    "Mysore" = "Mysore",
                    "Cachoeira" = "Cachoeira",
                    "Kananga" = "Kananga",
                    "Mauritius" = "Mauritius",
                    "Lovu" = "Lovu Fiji",
                    "Marajo" = "Marajo",
                    "Tyva Republic" = "Tyva Republic",
                    "Yasawa" = "Yasawa Fiji")

# plot
plot <- fitted |>
    ggplot(aes(x = FORMALED.mo,
               y = post_mean,
               group = indices,
               color = category))  +
    geom_line(alpha = 0.05) +
    viridis::scale_color_viridis(discrete = TRUE,
                                 option = "D",
                                 name = NULL,
                                 direction = -1,
                                 labels=c("Very rarely/never",
                                          "A few times per year",
                                          "A few times per month",
                                          "A few times per week",
                                          "Every day")) +
  guides(colour = guide_legend(override.aes = list(alpha = 1))) + #
   ↪  controls the legend alpha separately from the geom_line() alpha
  facet_wrap(~SITE, nrow = 3, ncol = 5, labeller =
   ↪  as_labeller(site_labels)) +
  scale_x_continuous(name="Years of formal education", breaks =
   ↪  c(0,10,20,30), labels = c("0","10","20","30")) +
  scale_y_continuous(name="Probability of each response option",
   ↪  breaks=c(0, .5, 1)) +
  theme(legend.position = c(0.92, 0.1),
        legend.text=element_text(size=7),
        legend.key=element_blank(),
        panel.grid.minor = element_blank(),
        panel.background = element_rect(fill = "white"),
        legend.background = element_rect(fill="transparent"))
```

```
  set.seed(NULL)

  return(plot)
}
```

```
g_comp_trend(m3mo, "BGTHINK") + ggtitle("How often do you think about
↪   [deity]?")
```

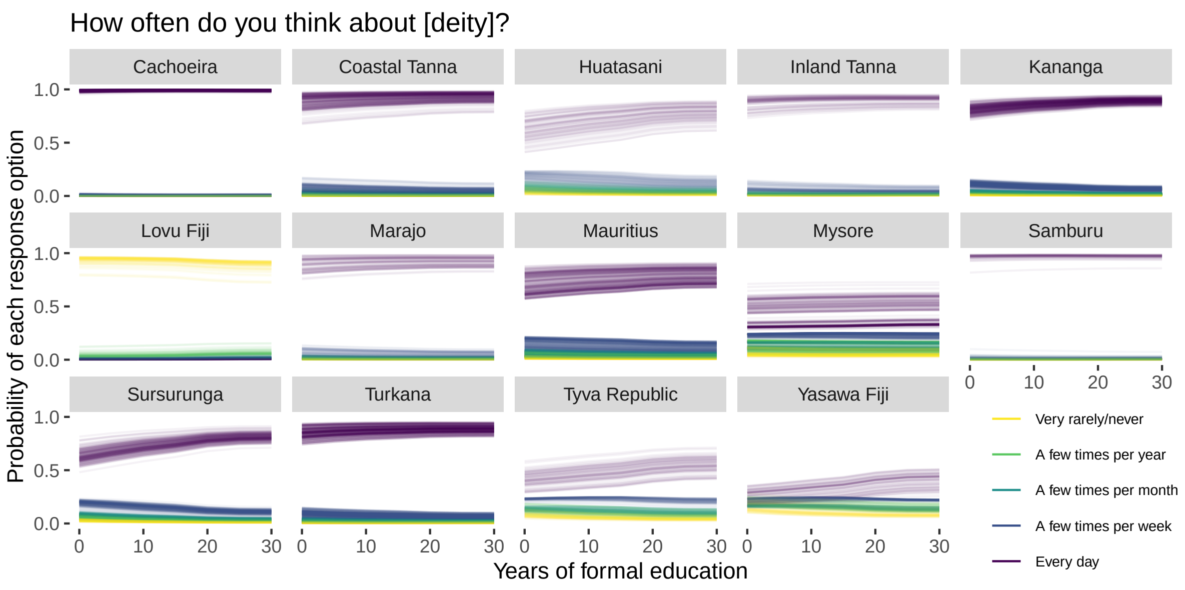


```
g_comp_trend(m3mo, "BGPERFHO") + ggtitle("How often do you perform
↪  activities or practices to talk to or appease [deity]?")
```

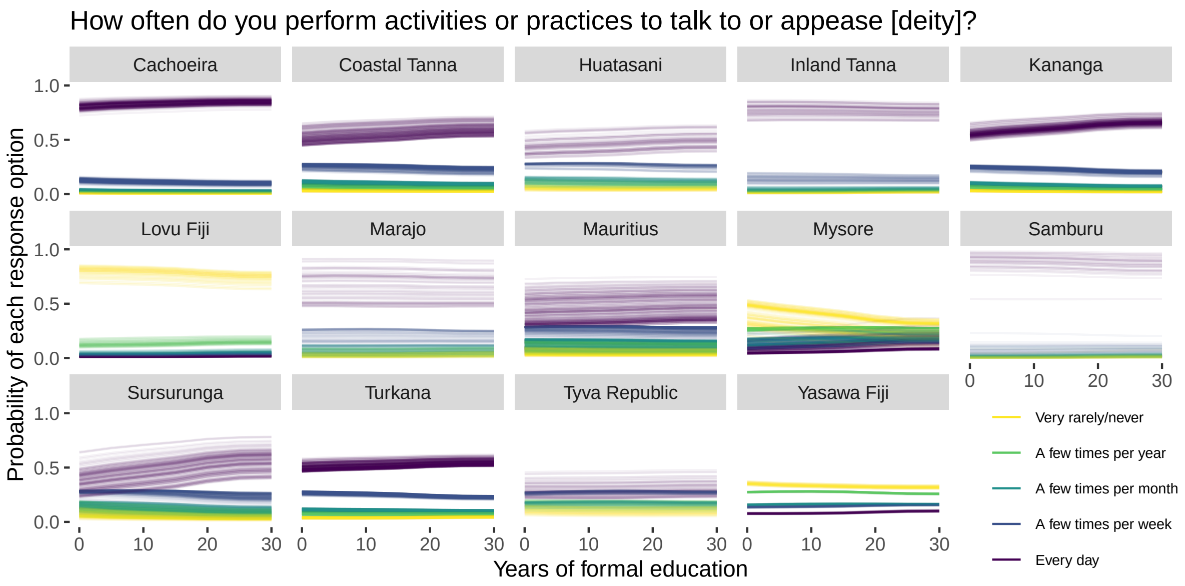

6 Multilevel Regression with Poststratification

Our main model and plotted predictions yield inferences for the sampled sites and participants with complete-cases for the included variables, when hypothetically manipulating years of formal education. While our dataset is both culturally and demographically diverse, it's not necessarily characteristic of any given population that we might be substantially interested in. Our results, then, potentially do not speak to any particular research question regarding any particular population.

For illustrative purposes, in this final section, we show how to go about obtaining predictions for a non-sampled but well-defined population, using so-called *poststratification*. Poststratification involves re-weighting a model's predictions using weights obtained from external data that are more representative of the population of interest.

For the sake of the illustration, we consider poststratifying model predictions to Denmark, since Danish (as well as many other European countries) census-level data are readily available for the relevant variables of age, gender, and educational level. We want to stress, however, that we do not take the obtained poststratified results as given but rather as a purely methodological exercise and practical demonstration.

The general steps in poststratification is as follows (see blocked-out comments in code chunks for more detail):

1) We obtain external data that are more representative of a population of interest, such as from a census. For the present analysis, we used Eurostat, a datahub for European census data: https://ec.europa.eu/eurostat/databrowser/view/EDAT_LFS_9901___custom_5352473/default/ta

2) We then load the external data and streamline the external data to our sample data set in terms of data structure, variables labels, etc.

```
## Construct external dataset to use for poststratification:
#
↪  https://ec.europa.eu/eurostat/databrowser/view/EDAT_LFS_9901__custom_5352473/default/t

# load external data for poststratification
eurostat <- read.csv("eurostat2021.csv", sep = ";")

psdf <- eurostat # copy df

# streamline name of education column
colnames(psdf)[which(names(psdf) == "ISCED11")] <- "FORMALED"

# bin demographic variables
psdf$SEX[psdf$SEX == "Females"] <- 0 # women
psdf$SEX[psdf$SEX == "Males"] <- 1 # men

psdf$AGE[psdf$AGE == "From 15 to 24 years"] <- 1 # 15-24
psdf$AGE[psdf$AGE == "From 25 to 34 years"] <- 2 # 25-34
psdf$AGE[psdf$AGE == "From 35 to 44 years"] <- 3 # 35-44
psdf$AGE[psdf$AGE == "From 45 to 54 years"] <- 4 # 45-54
psdf$AGE[psdf$AGE == "From 55 to 74 years"] <- 5 # 55-74

psdf$FORMALED[psdf$FORMALED == "Less than primary, primary and lower
↪  secondary education (levels 0-2)"] <- 1 # primary to lower secondary
psdf$FORMALED[psdf$FORMALED == "Upper secondary and post-secondary
↪  non-tertiary education (levels 3 and 4)"] <- 2 # # upper secondary
↪  and post-secondary non-tertiary education
psdf$FORMALED[psdf$FORMALED == "Tertiary education (levels 5-8)"] <- 3 #
↪  tertiary education
```

```
## Bin covariates according to Eurostat data
d_mrp <- d
```

```r
# to achieve maximal coverage of age groups while retaining reasonable
↪  (i.e., not too large, not too small) bin sizes
# consistent with externally available age ranges, we chose the
↪  following ranges:

d_mrp$AGE[d_mrp$AGE <= 24] <- 1 # below 24 (external age bin: 15-24)
d_mrp$AGE[d_mrp$AGE >= 25 & d_mrp$AGE <= 34] <- 2 # 25-34
d_mrp$AGE[d_mrp$AGE >= 35 & d_mrp$AGE <= 44] <- 3 # 35-44
d_mrp$AGE[d_mrp$AGE >= 45 & d_mrp$AGE <= 54] <- 4 # 45-54
d_mrp$AGE[d_mrp$AGE >= 55 & d_mrp$AGE <= 74] <- 5 # 55-74
d_mrp$AGE[d_mrp$AGE > 74] <- 6 # above 74; will not be used to
↪  poststratify due to lack of external data for this group

d_mrp$AGE <- as.factor(d_mrp$AGE) # convert to factor

d_mrp$FORMALED[d_mrp$FORMALED <= 10] <- 1 # ISCED 11: 0-2, primary to
↪  lower secondary, c. 10 years of education
d_mrp$FORMALED[d_mrp$FORMALED >= 11 & d_mrp$FORMALED <= 16] <- 2 # ISCED
↪  11: 3-4, upper secondary and post-secondary non-tertiary education,
↪  c. 5 years
d_mrp$FORMALED[d_mrp$FORMALED >= 16.1] <- 3 # ISCED 11: 5-8, tertiary
↪  education

d_mrp$FORMALED <- factor(d_mrp$FORMALED, ordered = TRUE) # convert to
↪  ordered factor
```

3) Next, we analyze our sample data in a multilevel cross-classification model that includes varying effects of the included demographic variables. Cross-classification address the fact that some cells (i.e., combination of covariates) might contain few observations and, through partial pooling, these cells are then informed by all other cells. As above, we plot simple prior vs. posterior predictive checks.

```r
mrppriors <- set_prior("normal(0,1)",
                        class = "b",
                        resp = "BGTHINK") +
              set_prior("normal(0,1)",
                        class = "b",
                        resp = "BGPERFHO") +
              set_prior("normal(0,2.5)",
                        class = "Intercept",
                        resp = "BGTHINK") +
```

```
                set_prior("normal(0,2.5)",
                          class = "Intercept",
                          resp = "BGPERFHO") +
                set_prior("dirichlet(2)",
                          class = "simo",
                          resp = "BGTHINK",
                          coef = "moFORMALED1") +
                set_prior("dirichlet(2)",
                          class = "simo",
                          resp = "BGPERFHO",
                          coef = "moFORMALED1") +
                set_prior("exponential(1)", class = "sd", resp =
                 ↪  "BGTHINK") +
                set_prior("exponential(1)", class = "sd", resp =
                 ↪  "BGPERFHO") +
                set_prior("lkj_corr_cholesky(4)", class = "L")

mrpformula <- mvbind(BGTHINK, BGPERFHO) ~ mo(FORMALED) + (mo(FORMALED) |
 ↪  SEX) + (mo(FORMALED) | AGE) + (mo(FORMALED) | SITE)

mrpppc <- fit_brms_ppc(formula = mrpformula, priors = mrppriors, data =
 ↪  d_mrp)

mrp <- fit_brms(formula = mrpformula, priors = mrppriors, data = d_mrp)
```

```
prior_vs_post(mrpppc, mrp)
```

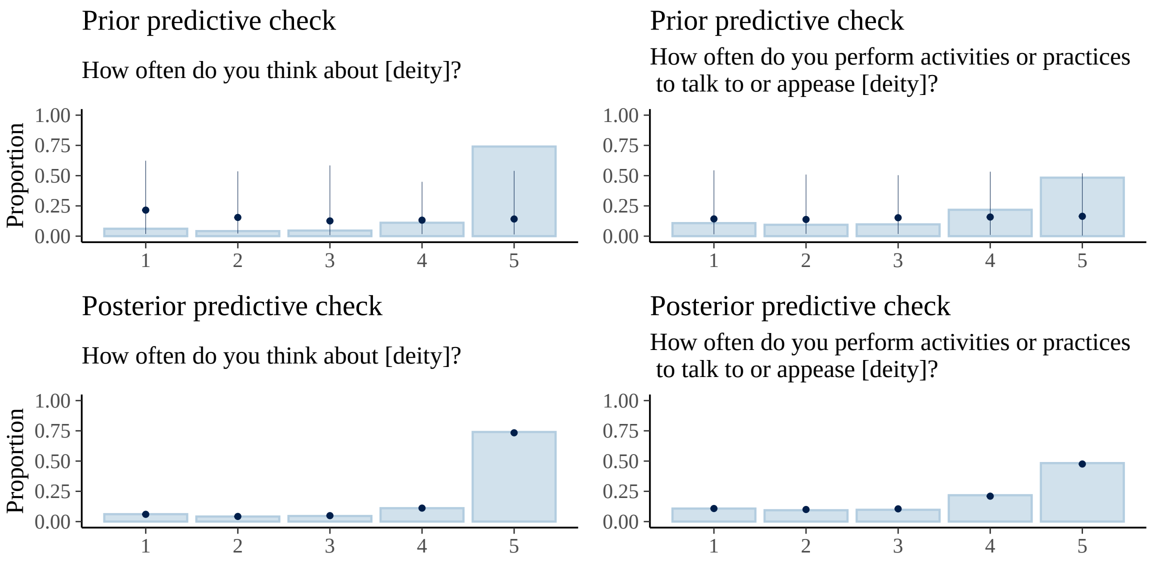

4) Then, we obtain poststratification weights from the external data. For each combination of covariates (gender, age, level of education), the weights are simply the proportion given by the number of Danes with this combination to the total number of Danes in the external data. These weights are then used to re-weigh the model predictions.

```
# get poststratification weights
ps_prop <- psdf  |>
  group_by(FORMALED) |>
  mutate(prop = Value/sum(Value)) |>
  ungroup()

# postratification function
poststratify <- function(model, newdata, resp) {
  set.seed(2021)

  # get predictions for new data
  ps_fitted <- add_epred_draws(
                  object = model,
                  newdata = newdata,
                  resp = resp,
                  ndraws = 300,

                  # excluding site-specific variation
```

```
                    re_formula = ~ (mo(FORMALED) | SEX) + (mo(FORMALED) |
                     ↪  AGE),
                    allow_new_levels = TRUE) |>

  # re-weight model predictions using proportions from above
  rename(estimate = .epred) |>
  mutate(estimate_prop = estimate*prop) |>
  group_by(FORMALED, .draw, .category) |>
  summarise(estimate_sum = sum(estimate_prop)) |>

  # connect predictions for line plotting
  group_by(.draw, .category) |>
  mutate(indices = cur_group_id()) |>
  ungroup() |>
  rename(.epred = estimate_sum)

  set.seed(NULL)

  return(ps_fitted)

}

ps_think <- poststratify(mrp, ps_prop, "BGTHINK")
ps_perfho <- poststratify(mrp, ps_prop, "BGPERFHO")
```

5) Finally, the re-weighted predictions are plotted across available levels of formal education for each outcome.

```
# plotting function
ps_gcomp_trend <- function(fitted){
  fitted |>
    ggplot(aes(x = FORMALED,
               y = .epred,
               group = indices,
               color = .category))  +
    geom_line(alpha = 0.05) +
    viridis::scale_color_viridis(discrete = TRUE,
                                 option = "D",
                                 name = NULL,
                                 direction = -1,
                                 labels=c("Very rarely/never",
```

```
                                            "A few times per year",
                                            "A few times per month",
                                            "A few times per week",
                                            "Every day")) +
  guides(colour = guide_legend(override.aes = list(alpha = 1))) + #
   ↪  controls the legend alpha separately from the geom_line() alpha
  scale_x_discrete(name="Years of formal education (binned)", breaks =
   ↪  c(1,2,3), labels = c("0-10","11-16",">16"),
                   expand = c(0,0.1)) +
  scale_y_continuous(name="Probability of each response option",
   ↪  breaks=c(0, .5, 1), limits = c(0,1)) +
  theme(strip.background = element_blank(),
        strip.text.x = element_blank(),
        panel.grid.minor = element_blank(),
        legend.key=element_blank(),
        panel.background = element_rect(fill = "white"))
}

# panel plotting
(ps_gcomp_trend(ps_think)) +
  theme(legend.position = "none") +
  labs(title = "Poststratified to Denmark",
       subtitle = "How often do you think about [deity]?") +
    (ps_gcomp_trend(ps_perfho)) +
      theme(axis.title.y = element_text(color = "white"),
            axis.text.y = element_blank()) +
      labs(title = "Poststratified to Denmark",
           subtitle = "How often do you perform activities or
            ↪  practices\n to talk to or appease [deity]?") +
  plot_layout(ncol = 2)
```

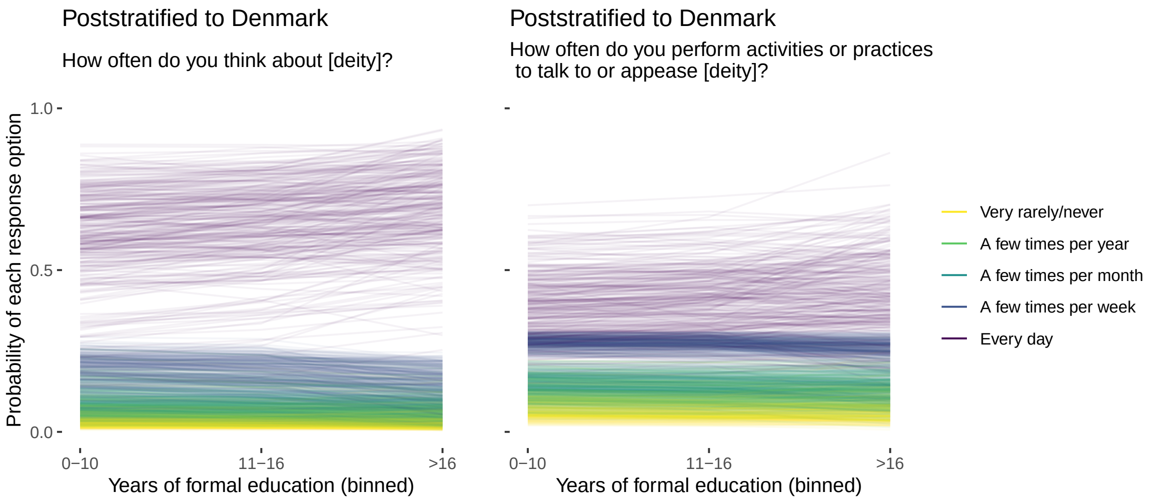

Now, notice that since years of formal education did not exhibit a clear effect on religious commitment in our sample data, the poststratified predictions are not qualitatively different from our main results. Consider too that our sample data were generally very religiously committed. Since the model is trained on this dataset, according to the poststratified predictions but counter to fact, Danes also come across as very religiously committed. We therefore stress again that, in the present study context, the poststratification exercise is purely a methodological illustration.

7 Identifiability assumptions

For our estimated effects to have valid causal interpretations, a set of identifiability assumptions need to be met.

First, the statistical model is assumed to not suffer from model misspecification, measurement bias, or unmodelled confounding (i.e., *conditional exchangeability*). Second, it's assumed that the exposure and outcome of participants do not depend on other participants' exposures and outcomes (i.e., *stable unit treatment value assumption* or *no interference*). Third, it's assumed that the observed outcome under a given exposure for any given participant is equivalent to the potential outcome that would've been observed for that participant under that exposure (i.e., *counterfactual consistency*). Fourth, it's assumed that all counterfactually manipulated levels and combinations of exposures and covariates are possible for all participants (i.e., *positivity*). This latter assumption can be relaxed to the extent that we're willing to extrapolate the model's predictions to ranges and combinations of exposures and covariates outside the observed sample. All these assumptions, however, are difficult or even impossible to verify using empirical data alone.

8 R packages, versions, and dependencies

We used R version 4.3.0 (R Core Team 2023) and the following R packages: abind v. 1.4.5 (Plate and Heiberger 2016), arrayhelpers v. 1.1.0 (Beleites 2020), backports v. 1.4.1 (Lang and R Core Team 2021), base64enc v. 0.1.3 (Urbanek 2015), bayesplot v. 1.10.0 (Gabry et al. 2019; Gabry and Mahr 2022), BH v. 1.81.0.1 (Eddelbuettel, Emerson, and Kane 2023), bridgesampling v. 1.1.2 (Gronau, Singmann, and Wagenmakers 2020), brms v. 2.19.0 (Bürkner 2017, 2018, 2021), Brobdingnag v. 1.2.9 (Hankin 2007), bslib v. 0.5.0 (Sievert, Cheng, and Aden-Buie 2023), cachem v. 1.0.8 (Chang 2023a), callr v. 3.7.3 (Csárdi and Chang 2022), checkmate v. 2.2.0 (Lang 2017), cmdstanr v. 0.5.3 (Gabry and Češnovar 2022), coda v. 0.19.4 (Plummer et al. 2006), colorspace v. 2.1.0 (Zeileis, Hornik, and Murrell 2009; Stauffer et al. 2009; Zeileis et al. 2020), colourpicker v. 1.2.0 (Attali 2022), commonmark v. 1.9.0 (Ooms 2023), cpp11 v. 0.4.3 (Hester and François 2022), crayon v. 1.5.2 (Csárdi 2022), crosstalk v. 1.2.0 (Cheng and Sievert 2021), desc v. 1.4.2 (Csárdi, Müller, and Hester 2022), digest v. 0.6.31 (Antoine Lucas et al. 2022), distributional v. 0.3.2 (O'Hara-Wild, Kay, and Hayes 2023), DT v. 0.28 (Xie, Cheng, and Tan 2023), dygraphs v. 1.1.1.6 (Vanderkam et al. 2018), ellipsis v. 0.3.2 (H. Wickham 2021), evaluate v. 0.21 (H. Wickham and Xie 2023), fansi v. 1.0.4 (Gaslam 2023), farver v. 2.1.1 (Pedersen, Nicolae, and François 2022), fastmap v. 1.1.1 (Chang 2023b), fontawesome v. 0.5.1 (Iannone 2023), fs v. 1.6.2 (Hester, Wickham, and Csárdi 2023), future v. 1.32.0 [@], generics v. 0.1.3 (H. Wickham, Kuhn, and Vaughan 2022), ggdist v. 3.3.0 (Kay 2023a), ggridges v. 0.5.4 (Wilke 2022), globals v. 0.16.2 (Bengtsson 2022a), glue v. 1.6.2 (Hester and Bryan 2022), gridExtra v. 2.3 (Auguie 2017), gtable v. 0.3.3 (H. Wickham and Pedersen 2023), gtools v. 3.9.4 (Bolker, Warnes, and Lumley 2022), highr v. 0.10 (Xie and Qiu 2022), htmltools v. 0.5.5 (Cheng, Sievert, et al. 2023), htmlwidgets v. 1.6.2 (Vaidyanathan et al. 2023), httpuv v. 1.6.11 (Cheng, Chang, et al. 2023), igraph v. 1.4.3 (Csardi and Nepusz 2006), inline v. 0.3.19 (Sklyar et al. 2021), isoband v. 0.2.7 (H. Wickham, Wilke, and Pedersen 2022), jquerylib v. 0.1.4 (Sievert and Cheng 2021), knitr v. 1.43 (Xie 2014, 2015, 2023a), labeling v. 0.4.2 (Justin Talbot 2020), later v. 1.3.1 (Chang and Cheng 2023), lazyeval v. 0.2.2 (H. Wickham 2019), lifecycle v. 1.0.3 (Henry and Wickham 2022a), listenv v. 0.9.0 (Bengtsson 2022b), loo v. 2.6.0 (Vehtari, Gelman, and Gabry 2017; Yao et al. 2017; Vehtari et al. 2023), markdown v. 1.10 (Xie, Allaire, and Horner 2023), matrixStats v. 1.0.0 (Bengtsson 2023a), memoise v. 2.0.1 (H. Wickham et al. 2021), mime v. 0.12 (Xie 2021), miniUI v. 0.1.1.1 (Cheng 2018), munsell v. 0.5.0 (C. Wickham 2018), mvtnorm v. 1.2.2 (Genz and Bretz 2009), nleqslv v. 3.3.4 (Hasselman 2023), numDeriv v. 2016.8.1.1 (Gilbert and Varadhan 2019), parallelly v. 1.36.0 (Bengtsson 2023b), patchwork v. 1.1.2 (Pedersen 2022), pkgbuild v. 1.4.1 (H. Wickham, Hester, and Csárdi 2023), pkgconfig v. 2.0.3 (Csárdi 2019), plyr v. 1.8.8 (H. Wickham 2011), posterior v. 1.4.1 (Vehtari et al. 2021; Bürkner et al. 2023), prettyunits v. 1.1.1 (Csardi 2020), processx v. 3.8.1 (Csárdi and Chang 2023), promises v. 1.2.0.1 (Cheng 2021), ps v. 1.7.5 (Loden et al. 2023), quadprog v. 1.5.8 (Berwin A. Turlach R port by Andreas Weingessel <Andreas.Weingessel@ci.tuwien.ac.at> Fortran contributions from Cleve Moler dpodi/LINPACK) 2019), QuickJSR v. 1.0.7 (Johnson 2023), R6 v. 2.5.1 (Chang 2021a), rappdirs v. 0.3.3 (Ratnakumar, Mick, and Davis 2021), RColorBrewer v.

1.1.3 (Neuwirth 2022), Rcpp v. 1.0.10 (Eddelbuettel and François 2011; Eddelbuettel 2013; Eddelbuettel and Balamuta 2018), RcppEigen v. 0.3.3.9.3 (Bates and Eddelbuettel 2013), RcppParallel v. 5.1.7 (Allaire, Francois, et al. 2023), remotes v. 2.4.2 (Csárdi et al. 2021), renv v. 1.0.0 (Ushey and Wickham 2023), reshape2 v. 1.4.4 (H. Wickham 2007), rmarkdown v. 2.22 (Xie, Allaire, and Grolemund 2018; Xie, Dervieux, and Riederer 2020; Allaire, Xie, et al. 2023), rprojroot v. 2.0.3 (Müller 2022), rstan v. 2.21.8 (Stan Development Team 2023), rstantools v. 2.3.1 (Gabry et al. 2023), sass v. 0.4.6 (Cheng, Mastny, et al. 2023), scales v. 1.2.1 (H. Wickham and Seidel 2022), shiny v. 1.7.4 (Chang et al. 2022), shinyjs v. 2.1.0 (Attali 2021), shinystan v. 2.6.0 (Gabry and Veen 2022), shinythemes v. 1.2.0 (Chang 2021b), sourcetools v. 0.1.7.1 (Ushey 2023), StanHeaders v. 2.26.27 (Stan Development Team 2020), stringi v. 1.7.12 (Gagolewski 2022), svUnit v. 1.0.6 (Grosjean 2023), tensorA v. 0.36.2 (van den Boogaart 2020), threejs v. 0.3.3 (Lewis 2020), tidybayes v. 3.0.4 (Kay 2023b), tidyselect v. 1.2.0 (Henry and Wickham 2022b), tidyverse v. 2.0.0 (H. Wickham et al. 2019), tinytex v. 0.45 (Xie 2019, 2023b), utf8 v. 1.2.3 (Perry 2023), vctrs v. 0.6.2 (H. Wickham, Henry, and Vaughan 2023), viridis v. 0.6.3 (Garnier et al. 2023a), viridisLite v. 0.4.2 (Garnier et al. 2023b), withr v. 2.5.0 (Hester et al. 2022), xfun v. 0.39 (Xie 2023c), xtable v. 1.8.4 (Dahl et al. 2019), xts v. 0.13.1 (Ryan and Ulrich 2023), yaml v. 2.3.7 (Garbett et al. 2023), zoo v. 1.8.12 (Zeileis and Grothendieck 2005).

9 References

Allaire, JJ, Romain Francois, Kevin Ushey, Gregory Vandenbrouck, Marcus Geelnard, and Intel. 2023. *RcppParallel: Parallel Programming Tools for "Rcpp"*. https://CRAN.R-project.org/package=RcppParallel.

Allaire, JJ, Yihui Xie, Christophe Dervieux, Jonathan McPherson, Javier Luraschi, Kevin Ushey, Aron Atkins, et al. 2023. *rmarkdown: Dynamic Documents for r*. https://github.com/rstudio/rmarkdown.

Antoine Lucas, Dirk Eddelbuettel with contributions by, Jarek Tuszynski, Henrik Bengtsson, Simon Urbanek, Mario Frasca, Bryan Lewis, Murray Stokely, et al. 2022. *digest: Create Compact Hash Digests of r Objects*. https://CRAN.R-project.org/package=digest.

Attali, Dean. 2021. *shinyjs: Easily Improve the User Experience of Your Shiny Apps in Seconds*. https://CRAN.R-project.org/package=shinyjs.

———. 2022. *colourpicker: A Colour Picker Tool for Shiny and for Selecting Colours in Plots*. https://CRAN.R-project.org/package=colourpicker.

Auguie, Baptiste. 2017. *gridExtra: Miscellaneous Functions for "Grid" Graphics*. https://CRAN.R-project.org/package=gridExtra.

Bates, Douglas, and Dirk Eddelbuettel. 2013. "Fast and Elegant Numerical Linear Algebra Using the RcppEigen Package." *Journal of Statistical Software* 52 (5): 1–24. https://doi.org/10.18637/jss.v052.i05.

Beleites, C. 2020. *arrayhelpers: Convenience Functions for Arrays*. https://CRAN.R-project.org/package=arrayhelpers.

Bengtsson, Henrik. 2022a. *globals: Identify Global Objects in r Expressions*. https://CRAN.R-project.org/package=globals.

———. 2022b. *listenv: Environments Behaving (Almost) as Lists*. https://CRAN.R-project.org/package=listenv.

———. 2023a. *matrixStats: Functions That Apply to Rows and Columns of Matrices (and to Vectors)*. https://CRAN.R-project.org/package=matrixStats.

———. 2023b. *parallelly: Enhancing the "parallel" Package*. https://CRAN.R-project.org/package=parallelly.

Berwin A. Turlach R port by Andreas Weingessel <Andreas.Weingessel@ci.tuwien.ac.at> Fortran contributions from Cleve Moler dpodi/LINPACK), S original by. 2019. *quadprog: Functions to Solve Quadratic Programming Problems*. https://CRAN.R-project.org/package=quadprog.

Bolker, Ben, Gregory R. Warnes, and Thomas Lumley. 2022. *gtools: Various r Programming Tools*. https://CRAN.R-project.org/package=gtools.

Bürkner, Paul-Christian. 2017. "brms: An R Package for Bayesian Multilevel Models Using Stan." *Journal of Statistical Software* 80 (1): 1–28. https://doi.org/10.18637/jss.v080.i01.

———. 2018. "Advanced Bayesian Multilevel Modeling with the R Package brms." *The R Journal* 10 (1): 395–411. https://doi.org/10.32614/RJ-2018-017.

———. 2021. "Bayesian Item Response Modeling in R with brms and Stan." *Journal of Statistical Software* 100 (5): 1–54. https://doi.org/10.18637/jss.v100.i05.

Bürkner, Paul-Christian, Jonah Gabry, Matthew Kay, and Aki Vehtari. 2023. "posterior: Tools for Working with Posterior Distributions." https://mc-stan.org/posterior/.

Chang, Winston. 2021a. *R6: Encapsulated Classes with Reference Semantics*. https://CRAN.R-project.org/package=R6.

———. 2021b. *shinythemes: Themes for Shiny*. https://CRAN.R-project.org/package=shinythemes.

———. 2023a. *cachem: Cache r Objects with Automatic Pruning*. https://CRAN.R-project.org/package=cachem.

———. 2023b. *fastmap: Fast Data Structures*. https://CRAN.R-project.org/package=fastmap.

Chang, Winston, and Joe Cheng. 2023. *later: Utilities for Scheduling Functions to Execute Later with Event Loops*. https://CRAN.R-project.org/package=later.

Chang, Winston, Joe Cheng, JJ Allaire, Carson Sievert, Barret Schloerke, Yihui Xie, Jeff Allen, Jonathan McPherson, Alan Dipert, and Barbara Borges. 2022. *shiny: Web Application Framework for r*. https://CRAN.R-project.org/package=shiny.

Cheng, Joe. 2018. *miniUI: Shiny UI Widgets for Small Screens*. https://CRAN.R-project.org/package=miniUI.

———. 2021. *promises: Abstractions for Promise-Based Asynchronous Programming*. https://CRAN.R-project.org/package=promises.

Cheng, Joe, Winston Chang, Steve Reid, James Brown, Bob Trower, and Alexander Peslyak. 2023. *httpuv: HTTP and WebSocket Server Library*. https://CRAN.R-project.org/package=httpuv.

Cheng, Joe, Timothy Mastny, Richard Iannone, Barret Schloerke, and Carson Sievert. 2023. *sass: Syntactically Awesome Style Sheets ("Sass")*. https://CRAN.R-project.org/package=sass.

Cheng, Joe, and Carson Sievert. 2021. *crosstalk: Inter-Widget Interactivity for HTML Widgets*. https://CRAN.R-project.org/package=crosstalk.

Cheng, Joe, Carson Sievert, Barret Schloerke, Winston Chang, Yihui Xie, and Jeff Allen. 2023. *htmltools: Tools for HTML*. https://CRAN.R-project.org/package=htmltools.

Csardi, Gabor. 2020. *prettyunits: Pretty, Human Readable Formatting of Quantities*. https://CRAN.R-project.org/package=prettyunits.

Csardi, Gabor, and Tamas Nepusz. 2006. "The Igraph Software Package for Complex Network Research." *InterJournal* Complex Systems: 1695. https://igraph.org.

Csárdi, Gábor. 2019. *pkgconfig: Private Configuration for "R" Packages*. https://CRAN.R-project.org/package=pkgconfig.

———. 2022. *crayon: Colored Terminal Output*. https://CRAN.R-project.org/package=crayon.

Csárdi, Gábor, and Winston Chang. 2022. *callr: Call r from r*. https://CRAN.R-project.org/package=callr.

———. 2023. *processx: Execute and Control System Processes*. https://CRAN.R-project.org/package=processx.

Csárdi, Gábor, Jim Hester, Hadley Wickham, Winston Chang, Martin Morgan, and Dan Tenenbaum. 2021. *remotes: R Package Installation from Remote Repositories, Including*

"GitHub". https://CRAN.R-project.org/package=remotes.

Csárdi, Gábor, Kirill Müller, and Jim Hester. 2022. *desc: Manipulate DESCRIPTION Files*. https://CRAN.R-project.org/package=desc.

Dahl, David B., David Scott, Charles Roosen, Arni Magnusson, and Jonathan Swinton. 2019. *xtable: Export Tables to LaTeX or HTML*. https://CRAN.R-project.org/package=xtable.

Eddelbuettel, Dirk. 2013. *Seamless R and C++ Integration with Rcpp*. New York: Springer. https://doi.org/10.1007/978-1-4614-6868-4.

Eddelbuettel, Dirk, and James Joseph Balamuta. 2018. "Extending extitR with extitC++: A Brief Introduction to extitRcpp." *The American Statistician* 72 (1): 28–36. https://doi.org/10.1080/00031305.2017.1375990.

Eddelbuettel, Dirk, John W. Emerson, and Michael J. Kane. 2023. *BH: Boost c++ Header Files*. https://CRAN.R-project.org/package=BH.

Eddelbuettel, Dirk, and Romain François. 2011. "Rcpp: Seamless R and C++ Integration." *Journal of Statistical Software* 40 (8): 1–18. https://doi.org/10.18637/jss.v040.i08.

Gabry, Jonah, and Rok Češnovar. 2022. *cmdstanr: R Interface to "CmdStan"*.

Gabry, Jonah, Ben Goodrich, Martin Lysy, and Andrew Johnson. 2023. *rstantools: Tools for Developing r Packages Interfacing with "Stan"*. https://CRAN.R-project.org/package=rstantools.

Gabry, Jonah, and Tristan Mahr. 2022. "bayesplot: Plotting for Bayesian Models." https://mc-stan.org/bayesplot/.

Gabry, Jonah, Daniel Simpson, Aki Vehtari, Michael Betancourt, and Andrew Gelman. 2019. "Visualization in Bayesian Workflow." *J. R. Stat. Soc. A* 182: 389–402. https://doi.org/10.1111/rssa.12378.

Gabry, Jonah, and Duco Veen. 2022. *shinystan: Interactive Visual and Numerical Diagnostics and Posterior Analysis for Bayesian Models*. https://CRAN.R-project.org/package=shinystan.

Gagolewski, Marek. 2022. "stringi: Fast and Portable Character String Processing in R." *Journal of Statistical Software* 103 (2): 1–59. https://doi.org/10.18637/jss.v103.i02.

Garbett, Shawn P, Jeremy Stephens, Kirill Simonov, Yihui Xie, Zhuoer Dong, Hadley Wickham, Jeffrey Horner, et al. 2023. *yaml: Methods to Convert r Data to YAML and Back*. https://CRAN.R-project.org/package=yaml.

Garnier, Simon, Ross, Noam, Rudis, Robert, Camargo, et al. 2023a. *viridis(Lite) - Colorblind-Friendly Color Maps for r*. https://doi.org/10.5281/zenodo.4679424.

———, et al. 2023b. *viridis(Lite) - Colorblind-Friendly Color Maps for r*. https://doi.org/10.5281/zenodo.4678327.

Gaslam, Brodie. 2023. *fansi: ANSI Control Sequence Aware String Functions*. https://CRAN.R-project.org/package=fansi.

Genz, Alan, and Frank Bretz. 2009. *Computation of Multivariate Normal and t Probabilities*. Lecture Notes in Statistics. Heidelberg: Springer-Verlag.

Gilbert, Paul, and Ravi Varadhan. 2019. *numDeriv: Accurate Numerical Derivatives*. https://CRAN.R-project.org/package=numDeriv.

Gronau, Quentin F., Henrik Singmann, and Eric-Jan Wagenmakers. 2020. "bridgesampling:

An R Package for Estimating Normalizing Constants." *Journal of Statistical Software* 92 (10): 1–29. https://doi.org/10.18637/jss.v092.i10.

Grosjean, Philippe. 2023. *SciViews-r*. MONS, Belgium: UMONS. https://www.sciviews.org/SciViews-R/.

Hankin, R. K. S. 2007. "Very Large Numbers in r: Introducing Package Brobdingnag." *R News* 7.

Hasselman, Berend. 2023. *nleqslv: Solve Systems of Nonlinear Equations*. https://CRAN.R-project.org/package=nleqslv.

Henry, Lionel, and Hadley Wickham. 2022a. *lifecycle: Manage the Life Cycle of Your Package Functions*. https://CRAN.R-project.org/package=lifecycle.

———. 2022b. *tidyselect: Select from a Set of Strings*. https://CRAN.R-project.org/package=tidyselect.

Hester, Jim, and Jennifer Bryan. 2022. *glue: Interpreted String Literals*. https://CRAN.R-project.org/package=glue.

Hester, Jim, and Romain François. 2022. *Cpp11: A c++11 Interface for r's c Interface*. https://CRAN.R-project.org/package=cpp11.

Hester, Jim, Lionel Henry, Kirill Müller, Kevin Ushey, Hadley Wickham, and Winston Chang. 2022. *withr: Run Code "With" Temporarily Modified Global State*. https://CRAN.R-project.org/package=withr.

Hester, Jim, Hadley Wickham, and Gábor Csárdi. 2023. *fs: Cross-Platform File System Operations Based on "libuv"*. https://CRAN.R-project.org/package=fs.

Iannone, Richard. 2023. *fontawesome: Easily Work with "Font Awesome" Icons*. https://CRAN.R-project.org/package=fontawesome.

Johnson, Andrew R. 2023. *QuickJSR: Interface for the "QuickJS" Lightweight "JavaScript" Engine*. https://CRAN.R-project.org/package=QuickJSR.

Justin Talbot. 2020. *labeling: Axis Labeling*. https://CRAN.R-project.org/package=labeling.

Kay, Matthew. 2023a. *ggdist: Visualizations of Distributions and Uncertainty*. https://doi.org/10.5281/zenodo.3879620.

———. 2023b. *tidybayes: Tidy Data and Geoms for Bayesian Models*. https://doi.org/10.5281/zenodo.1308151.

Lang, Michel. 2017. "checkmate: Fast Argument Checks for Defensive R Programming." *The R Journal* 9 (1): 437–45. https://doi.org/10.32614/RJ-2017-028.

Lang, Michel, and R Core Team. 2021. *backports: Reimplementations of Functions Introduced Since r-3.0.0*. https://CRAN.R-project.org/package=backports.

Lewis, B. W. 2020. *threejs: Interactive 3D Scatter Plots, Networks and Globes*. https://CRAN.R-project.org/package=threejs.

Loden, Jay, Dave Daeschler, Giampaolo Rodola', and Gábor Csárdi. 2023. *ps: List, Query, Manipulate System Processes*. https://CRAN.R-project.org/package=ps.

Müller, Kirill. 2022. *rprojroot: Finding Files in Project Subdirectories*. https://CRAN.R-project.org/package=rprojroot.

Neuwirth, Erich. 2022. *RColorBrewer: ColorBrewer Palettes*. https://CRAN.R-project.org/package=RColorBrewer.

O'Hara-Wild, Mitchell, Matthew Kay, and Alex Hayes. 2023. *distributional: Vectorised Probability Distributions*. https://CRAN.R-project.org/package=distributional.

Ooms, Jeroen. 2023. *commonmark: High Performance CommonMark and Github Markdown Rendering in r*. https://CRAN.R-project.org/package=commonmark.

Pedersen, Thomas Lin. 2022. *patchwork: The Composer of Plots*. https://CRAN.R-project.org/package=patchwork.

Pedersen, Thomas Lin, Berendea Nicolae, and Romain François. 2022. *farver: High Performance Colour Space Manipulation*. https://CRAN.R-project.org/package=farver.

Perry, Patrick O. 2023. *Utf8: Unicode Text Processing*. https://CRAN.R-project.org/package=utf8.

Plate, Tony, and Richard Heiberger. 2016. *abind: Combine Multidimensional Arrays*. https://CRAN.R-project.org/package=abind.

Plummer, Martyn, Nicky Best, Kate Cowles, and Karen Vines. 2006. "CODA: Convergence Diagnosis and Output Analysis for MCMC." *R News* 6 (1): 7–11. https://journal.r-project.org/archive/.

R Core Team. 2023. *R: A Language and Environment for Statistical Computing*. Vienna, Austria: R Foundation for Statistical Computing. https://www.R-project.org/.

Ratnakumar, Sridhar, Trent Mick, and Trevor Davis. 2021. *rappdirs: Application Directories: Determine Where to Save Data, Caches, and Logs*. https://CRAN.R-project.org/package=rappdirs.

Ryan, Jeffrey A., and Joshua M. Ulrich. 2023. *xts: eXtensible Time Series*. https://CRAN.R-project.org/package=xts.

Sievert, Carson, and Joe Cheng. 2021. *jquerylib: Obtain "jQuery" as an HTML Dependency Object*. https://CRAN.R-project.org/package=jquerylib.

Sievert, Carson, Joe Cheng, and Garrick Aden-Buie. 2023. *bslib: Custom "Bootstrap" "Sass" Themes for "shiny" and "rmarkdown"*. https://CRAN.R-project.org/package=bslib.

Sklyar, Oleg, Duncan Murdoch, Mike Smith, Dirk Eddelbuettel, Romain Francois, Karline Soetaert, and Johannes Ranke. 2021. *inline: Functions to Inline c, c++, Fortran Function Calls from r*. https://CRAN.R-project.org/package=inline.

Stan Development Team. 2020. "StanHeaders: Headers for the R Interface to Stan." https://mc-stan.org/.

———. 2023. "RStan: The R Interface to Stan." https://mc-stan.org/.

Stauffer, Reto, Georg J. Mayr, Markus Dabernig, and Achim Zeileis. 2009. "Somewhere over the Rainbow: How to Make Effective Use of Colors in Meteorological Visualizations." *Bulletin of the American Meteorological Society* 96 (2): 203–16. https://doi.org/10.1175/BAMS-D-13-00155.1.

Urbanek, Simon. 2015. *Base64enc: Tools for Base64 Encoding*. https://CRAN.R-project.org/package=base64enc.

Ushey, Kevin. 2023. *sourcetools: Tools for Reading, Tokenizing and Parsing r Code*. https://CRAN.R-project.org/package=sourcetools.

Ushey, Kevin, and Hadley Wickham. 2023. *renv: Project Environments*. https://CRAN.R-project.org/package=renv.

Vaidyanathan, Ramnath, Yihui Xie, JJ Allaire, Joe Cheng, Carson Sievert, and Kenton Russell.

2023. *htmlwidgets: HTML Widgets for r*. https://CRAN.R-project.org/package=htmlwidgets.

van den Boogaart, K. Gerald. 2020. *tensorA: Advanced Tensor Arithmetic with Named Indices*. https://CRAN.R-project.org/package=tensorA.

Vanderkam, Dan, JJ Allaire, Jonathan Owen, Daniel Gromer, and Benoit Thieurmel. 2018. *dygraphs: Interface to "Dygraphs" Interactive Time Series Charting Library*. https://CRAN.R-project.org/package=dygraphs.

Vehtari, Aki, Jonah Gabry, Mans Magnusson, Yuling Yao, Paul-Christian Bürkner, Topi Paananen, and Andrew Gelman. 2023. "loo: Efficient Leave-One-Out Cross-Validation and WAIC for Bayesian Models." https://mc-stan.org/loo/.

Vehtari, Aki, Andrew Gelman, and Jonah Gabry. 2017. "Practical Bayesian Model Evaluation Using Leave-One-Out Cross-Validation and WAIC." *Statistics and Computing* 27: 1413–32. https://doi.org/10.1007/s11222-016-9696-4.

Vehtari, Aki, Andrew Gelman, Daniel Simpson, Bob Carpenter, and Paul-Christian Bürkner. 2021. "Rank-Normalization, Folding, and Localization: An Improved Rhat for Assessing Convergence of MCMC (with Discussion)." *Bayesian Analysis*.

Wickham, Charlotte. 2018. *munsell: Utilities for Using Munsell Colours*. https://CRAN.R-project.org/package=munsell.

Wickham, Hadley. 2007. "Reshaping Data with the reshape Package." *Journal of Statistical Software* 21 (12): 1–20. http://www.jstatsoft.org/v21/i12/.

———. 2011. "The Split-Apply-Combine Strategy for Data Analysis." *Journal of Statistical Software* 40 (1): 1–29. https://www.jstatsoft.org/v40/i01/.

———. 2019. *lazyeval: Lazy (Non-Standard) Evaluation*. https://CRAN.R-project.org/package=lazyeval.

———. 2021. *ellipsis: Tools for Working with …* https://CRAN.R-project.org/package=ellipsis.

Wickham, Hadley, Mara Averick, Jennifer Bryan, Winston Chang, Lucy D'Agostino McGowan, Romain François, Garrett Grolemund, et al. 2019. "Welcome to the tidyverse." *Journal of Open Source Software* 4 (43): 1686. https://doi.org/10.21105/joss.01686.

Wickham, Hadley, Lionel Henry, and Davis Vaughan. 2023. *vctrs: Vector Helpers*. https://CRAN.R-project.org/package=vctrs.

Wickham, Hadley, Jim Hester, Winston Chang, Kirill Müller, and Daniel Cook. 2021. *memoise: "Memoisation" of Functions*. https://CRAN.R-project.org/package=memoise.

Wickham, Hadley, Jim Hester, and Gábor Csárdi. 2023. *pkgbuild: Find Tools Needed to Build r Packages*. https://CRAN.R-project.org/package=pkgbuild.

Wickham, Hadley, Max Kuhn, and Davis Vaughan. 2022. *generics: Common S3 Generics Not Provided by Base r Methods Related to Model Fitting*. https://CRAN.R-project.org/package=generics.

Wickham, Hadley, and Thomas Lin Pedersen. 2023. *gtable: Arrange "Grobs" in Tables*. https://CRAN.R-project.org/package=gtable.

Wickham, Hadley, and Dana Seidel. 2022. *scales: Scale Functions for Visualization*. https://CRAN.R-project.org/package=scales.

Wickham, Hadley, Claus O. Wilke, and Thomas Lin Pedersen. 2022. *isoband: Generate*

Isolines and Isobands from Regularly Spaced Elevation Grids. https://CRAN.R-project.org/package=isoband.

Wickham, Hadley, and Yihui Xie. 2023. *evaluate: Parsing and Evaluation Tools That Provide More Details Than the Default*. https://CRAN.R-project.org/package=evaluate.

Wilke, Claus O. 2022. *ggridges: Ridgeline Plots in "ggplot2"*. https://CRAN.R-project.org/package=ggridges.

Xie, Yihui. 2014. "knitr: A Comprehensive Tool for Reproducible Research in R." In *Implementing Reproducible Computational Research*, edited by Victoria Stodden, Friedrich Leisch, and Roger D. Peng. Chapman; Hall/CRC.

———. 2015. *Dynamic Documents with R and Knitr*. 2nd ed. Boca Raton, Florida: Chapman; Hall/CRC. https://yihui.org/knitr/.

———. 2019. "TinyTeX: A Lightweight, Cross-Platform, and Easy-to-Maintain LaTeX Distribution Based on TeX Live." *TUGboat* 40 (1): 30–32. https://tug.org/TUGboat/Contents/contents40-1.html.

———. 2021. *mime: Map Filenames to MIME Types*. https://CRAN.R-project.org/package=mime.

———. 2023a. *knitr: A General-Purpose Package for Dynamic Report Generation in r*. https://yihui.org/knitr/.

———. 2023b. *tinytex: Helper Functions to Install and Maintain TeX Live, and Compile LaTeX Documents*. https://github.com/rstudio/tinytex.

———. 2023c. *xfun: Supporting Functions for Packages Maintained by "Yihui Xie"*. https://CRAN.R-project.org/package=xfun.

Xie, Yihui, J. J. Allaire, and Garrett Grolemund. 2018. *R Markdown: The Definitive Guide*. Boca Raton, Florida: Chapman; Hall/CRC. https://bookdown.org/yihui/rmarkdown.

Xie, Yihui, JJ Allaire, and Jeffrey Horner. 2023. *markdown: Render Markdown with "commonmark"*. https://CRAN.R-project.org/package=markdown.

Xie, Yihui, Joe Cheng, and Xianying Tan. 2023. *DT: A Wrapper of the JavaScript Library "DataTables"*. https://CRAN.R-project.org/package=DT.

Xie, Yihui, Christophe Dervieux, and Emily Riederer. 2020. *R Markdown Cookbook*. Boca Raton, Florida: Chapman; Hall/CRC. https://bookdown.org/yihui/rmarkdown-cookbook.

Xie, Yihui, and Yixuan Qiu. 2022. *highr: Syntax Highlighting for r Source Code*. https://CRAN.R-project.org/package=highr.

Yao, Yuling, Aki Vehtari, Daniel Simpson, and Andrew Gelman. 2017. "Using Stacking to Average Bayesian Predictive Distributions." *Bayesian Analysis*. https://doi.org/10.1214/17-BA1091.

Zeileis, Achim, Jason C. Fisher, Kurt Hornik, Ross Ihaka, Claire D. McWhite, Paul Murrell, Reto Stauffer, and Claus O. Wilke. 2020. "colorspace: A Toolbox for Manipulating and Assessing Colors and Palettes." *Journal of Statistical Software* 96 (1): 1–49. https://doi.org/10.18637/jss.v096.i01.

Zeileis, Achim, and Gabor Grothendieck. 2005. "zoo: S3 Infrastructure for Regular and Irregular Time Series." *Journal of Statistical Software* 14 (6): 1–27. https://doi.org/10.18637/jss.v014.i06.

Zeileis, Achim, Kurt Hornik, and Paul Murrell. 2009. "Escaping RGBland: Selecting Colors for Statistical Graphics." *Computational Statistics & Data Analysis* 53 (9): 3259–70. https://doi.org/10.1016/j.csda.2008.11.033.
